# Supplementary material for: Daphnane-Type Diterpenes from Stelleropsis tianschanica and Their Antitumor Activity
Source: Molecules. 2022 Sep 4;27(17):5701. doi: 10.3390/molecules27175701 (PMC9458044; doi:10.3390/molecules27175701)
Supplement: Supplementary file 1 [file molecules-27-05701-s001.zip › Supporting Information.pdf]

Supporting Information

Daphnane-type Diterpenes from *Stelleropsis tianschanica* and Their Anti-tumor Activity

Xiaoyan He [1,†], Xiatiguli·Abulizi [2,†], Xiaowan Li [3], Guoxu Ma [2,4,†], Zhaocui Sun [4], Hongyan Wei [2], Xudong Xu [4], Leiling Shi [2,*] and Jing Zhang [1,*]

[1] College of Chinese Medicine Material, Jilin Agricultural University, Changchun 130118, China;

[2] Xinjiang Institute of Chinese and Ethnic Medicine, Urumqi 830002, China;

[3] Xinjiang Agricultural Vocational and Technical College Biological Technology Branch, Changji 831100, China;

[4] Institute of Medicinal Plant Development, Chinese Academy of Medical Sciences and Peking Union Medical College, Beijing 100193, China

* Correspondence: shileiling163@163.com (L. L. S.); zhjing0701@163.com (J. Z.)

† These authors contributed equally to this work.

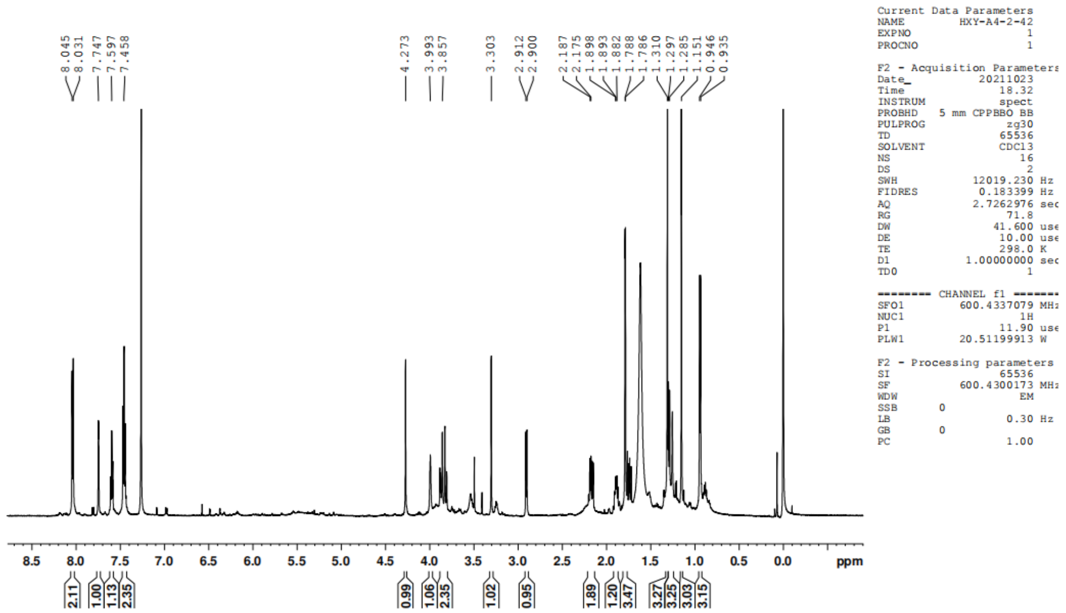

Figure S1. ^{1}H-NMR (600 MHz, $CDCl_3$) spectrum of **1**

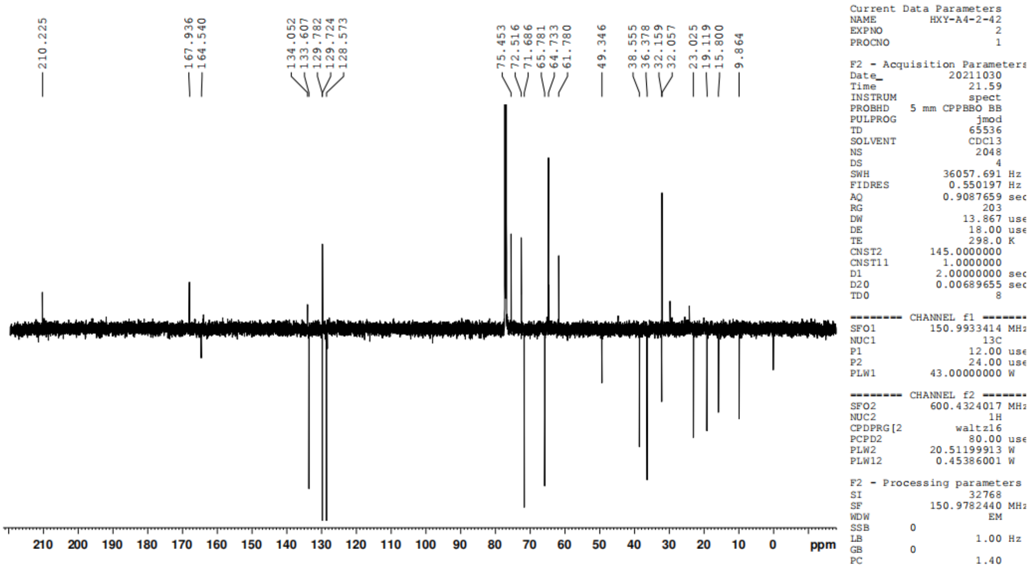

Figure S2. ^{13}C-APT (150 MHz, $CDCl_3$) spectrum of **1**

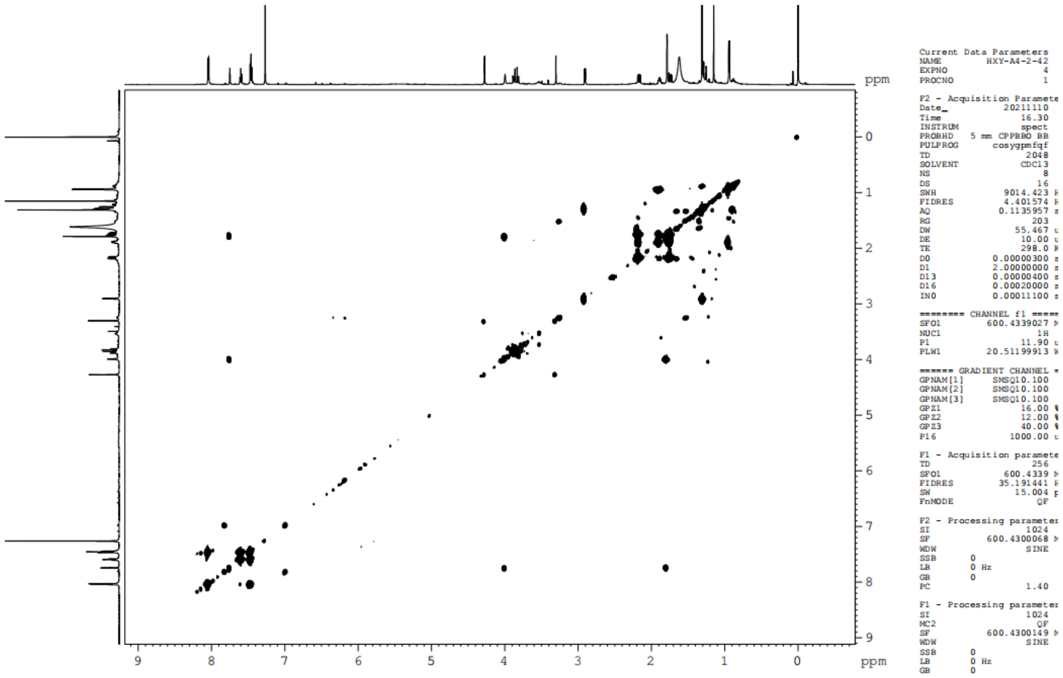

Figure S3. ^{1}H-^{1}H COSY ($CDCl_3$) spectrum of **1**

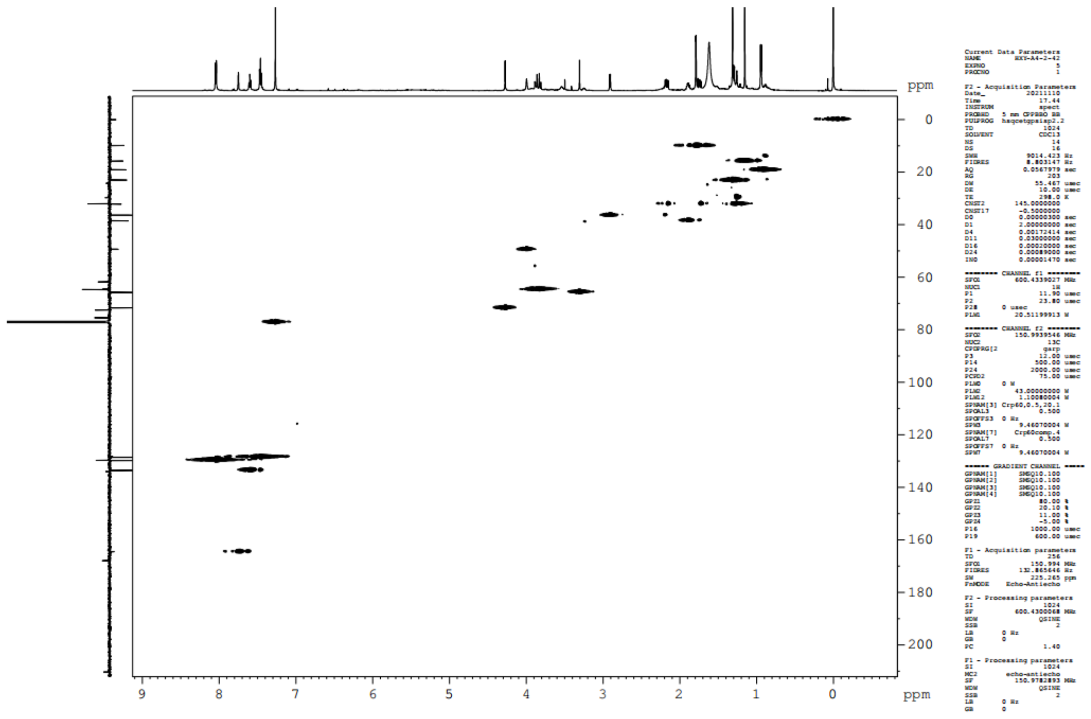

Figure S4. HSQC ($CDCl_3$) spectrum of **1**

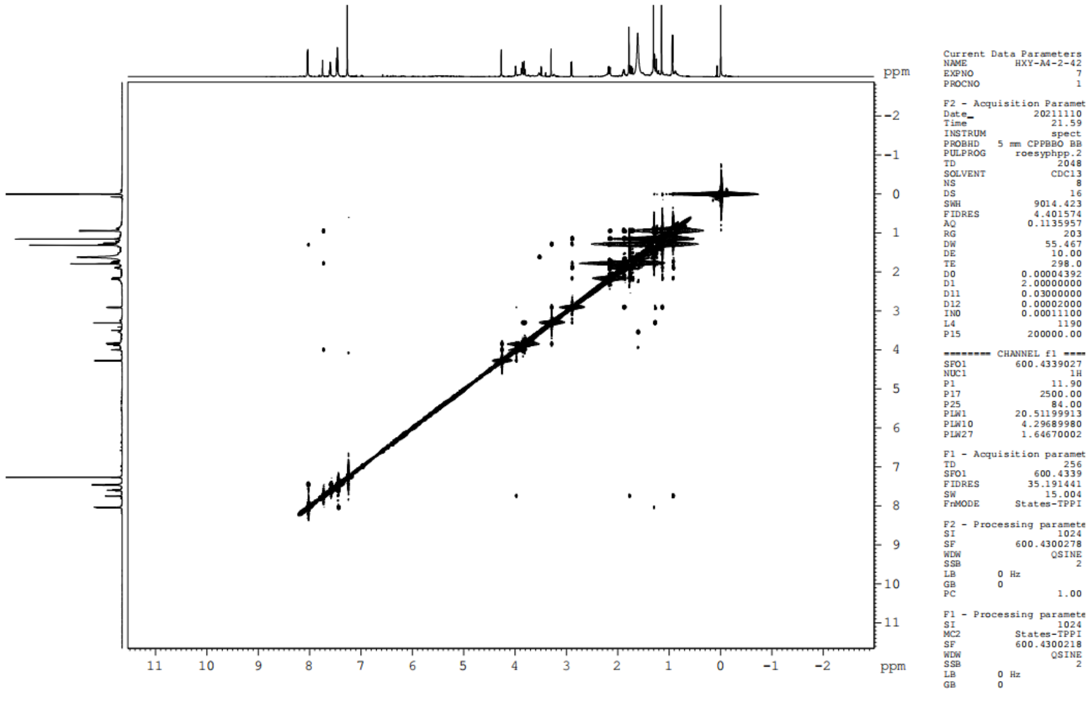

Figure S5. NOESY ($CDCl_3$) spectrum of **1**

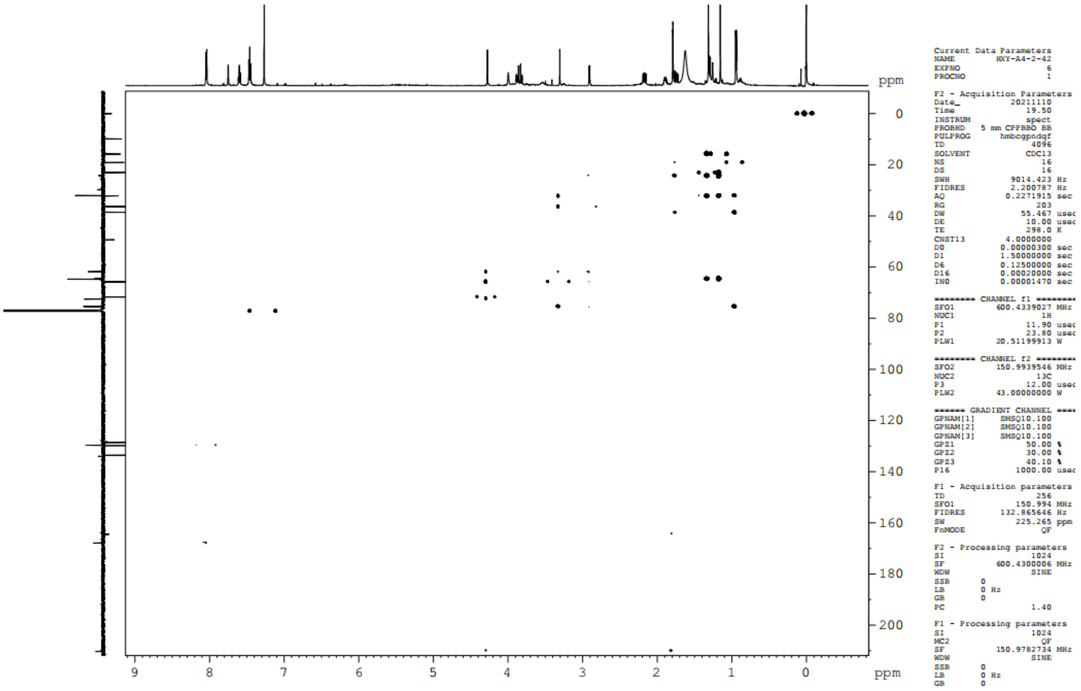

Figure S6. HMBC ($CDCl_3$) spectrum of **1**

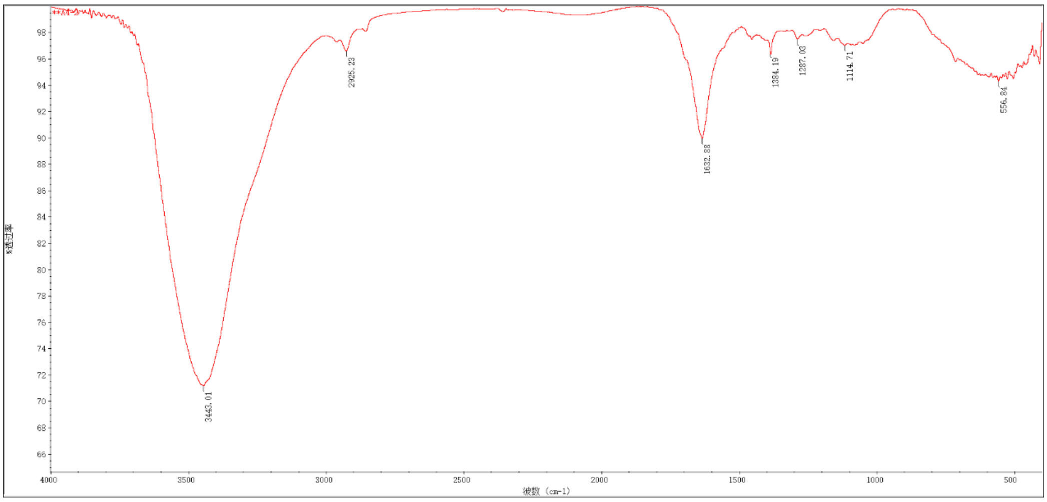

Figure S7. IR spectrum of **1**

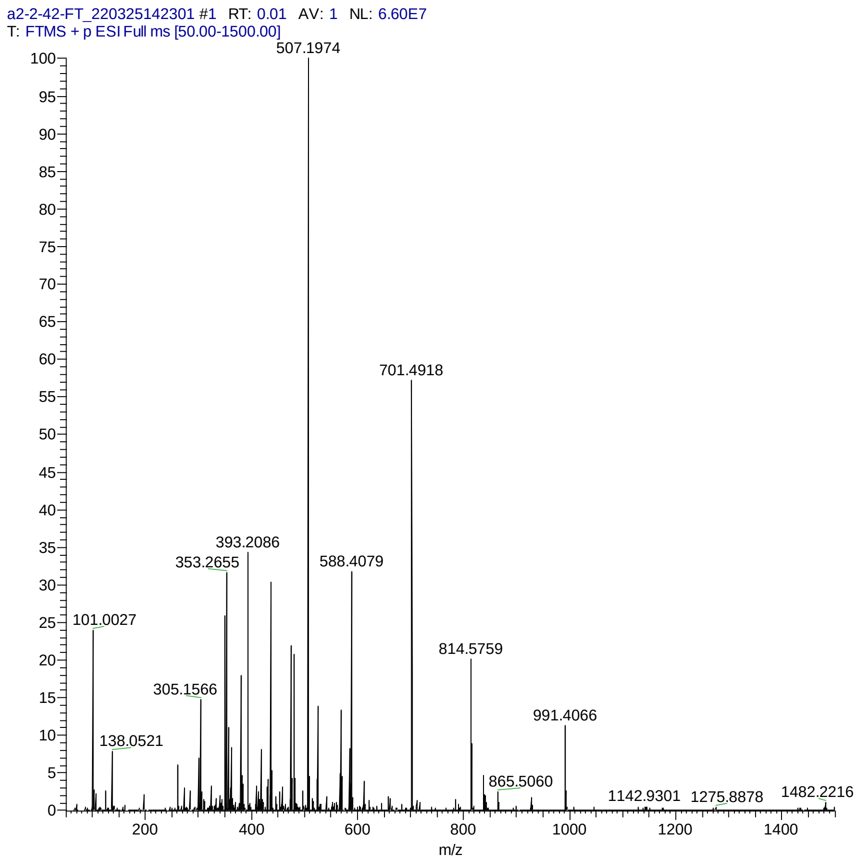

Figure S8. HRESIMS spectrum of **1**

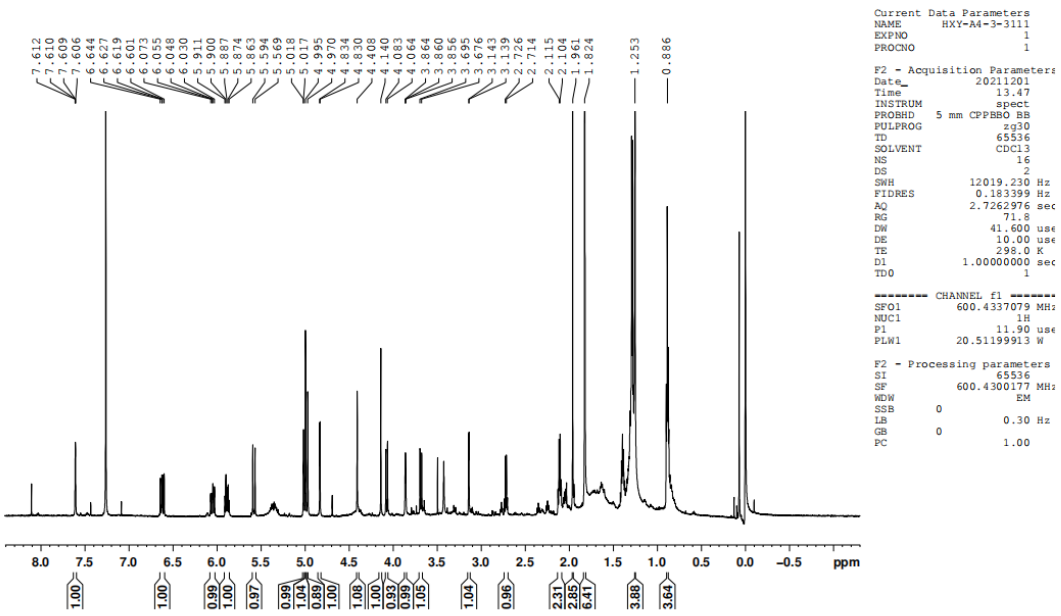

Figure S9. ^{1}H-NMR (600 MHz, $CDCl_3$) spectrum of **2**

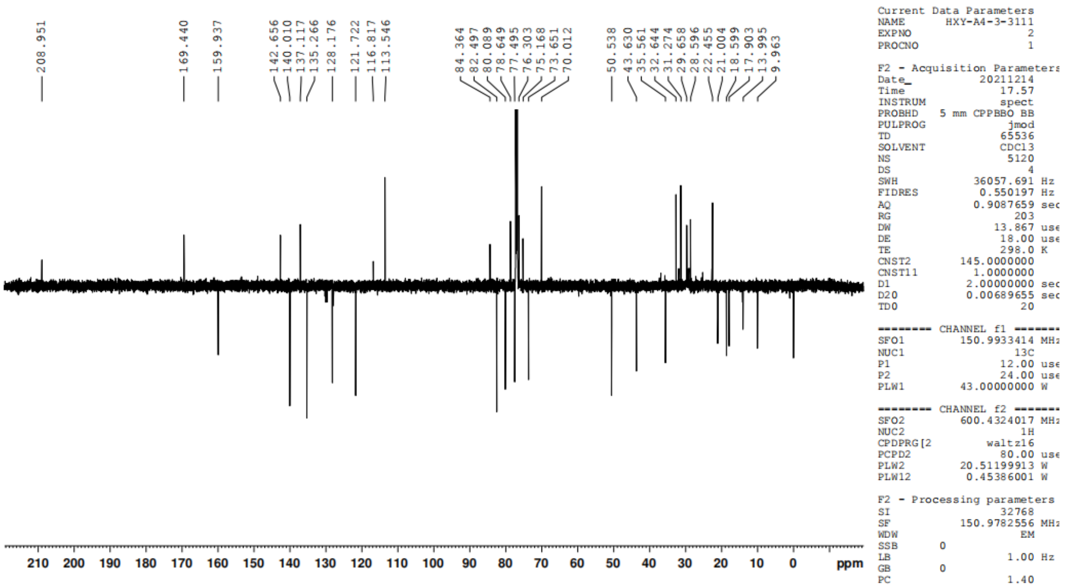

Figure S10. ^{13}C-APT (150 MHz, $CDCl_3$) spectrum of **2**

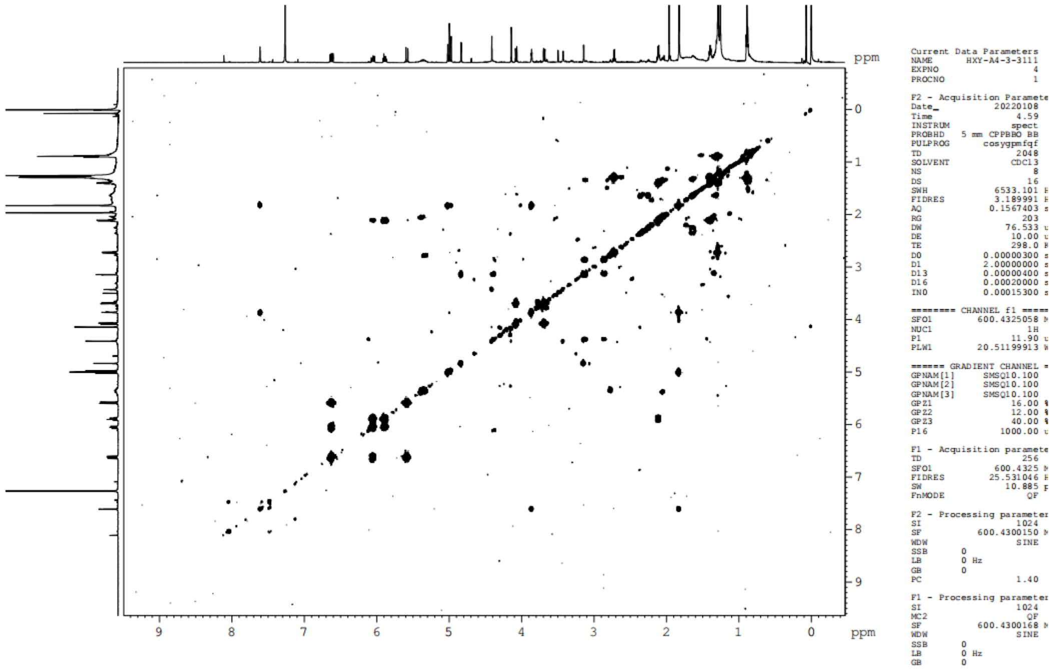

Figure S11. ^{1}H-^{1}H COSY ($CDCl_3$) spectrum of **2**

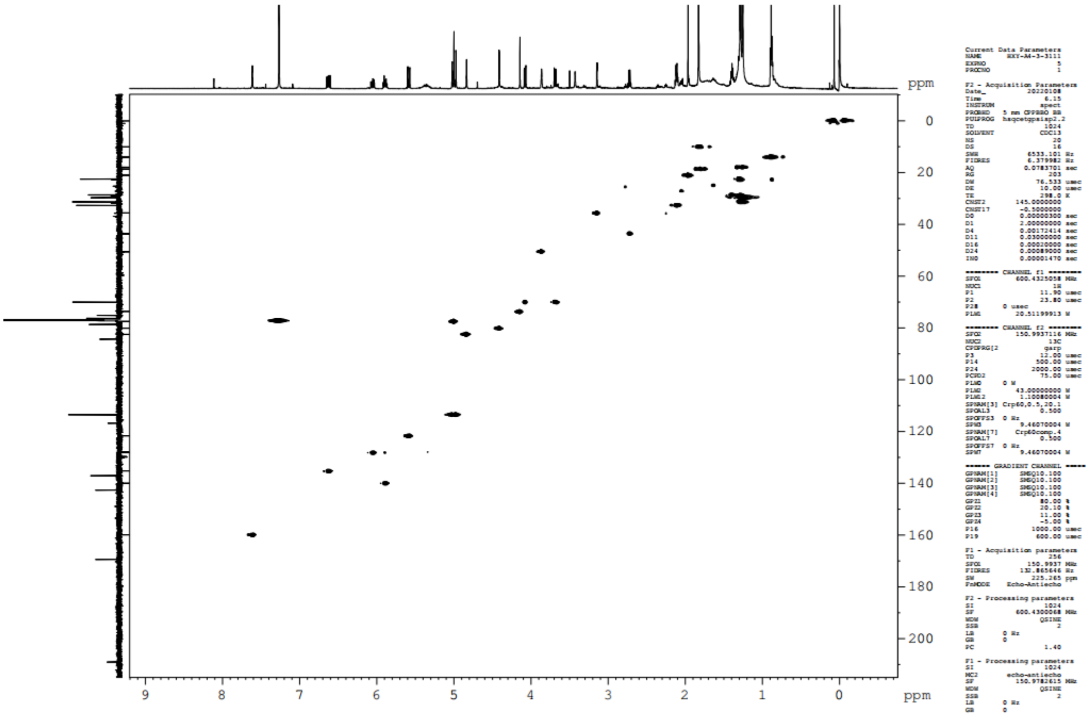

Figure S12. HSQC ($CDCl_3$) spectrum of **2**

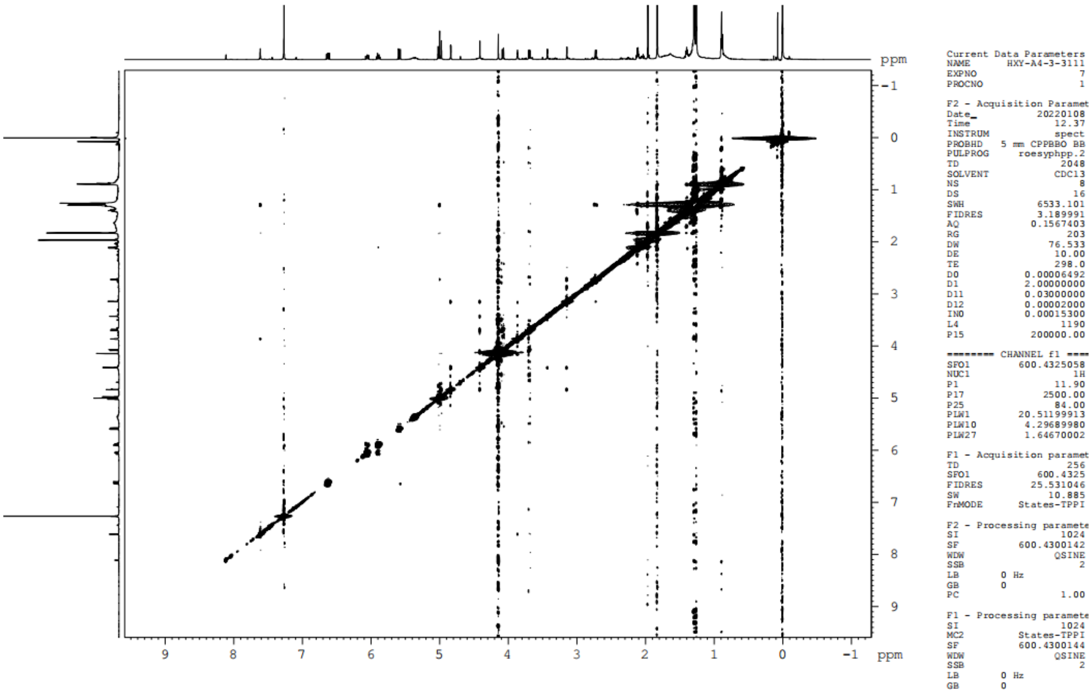

Figure S13. NOESY ($CDCl_3$) spectrum of **2**

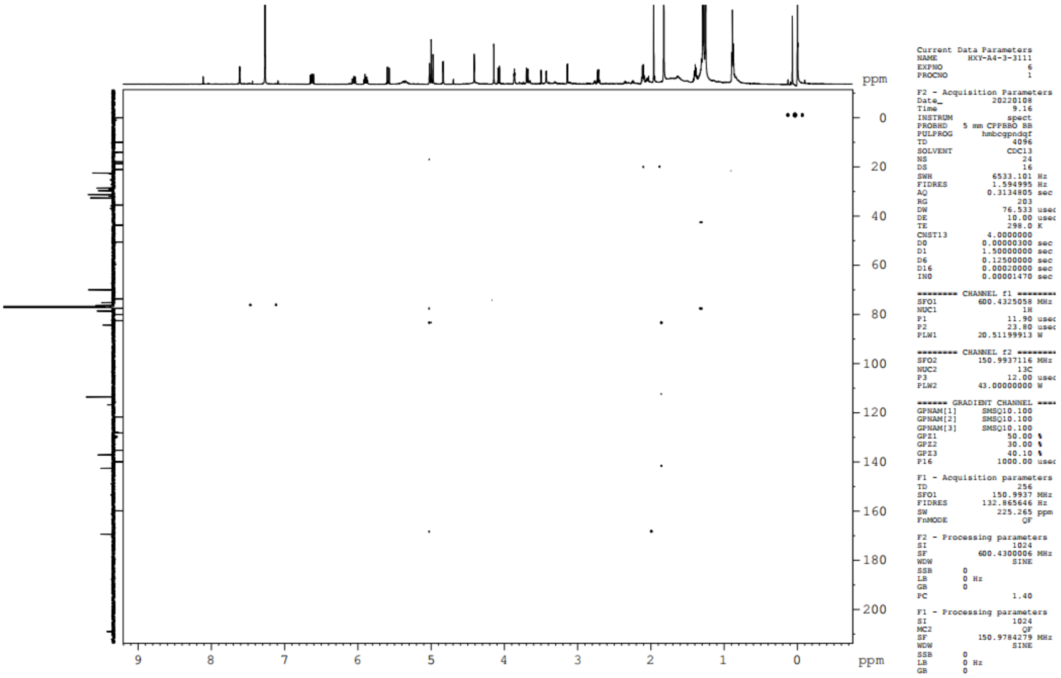

Figure S14. HMBC ($CDCl_3$) spectrum of **2**

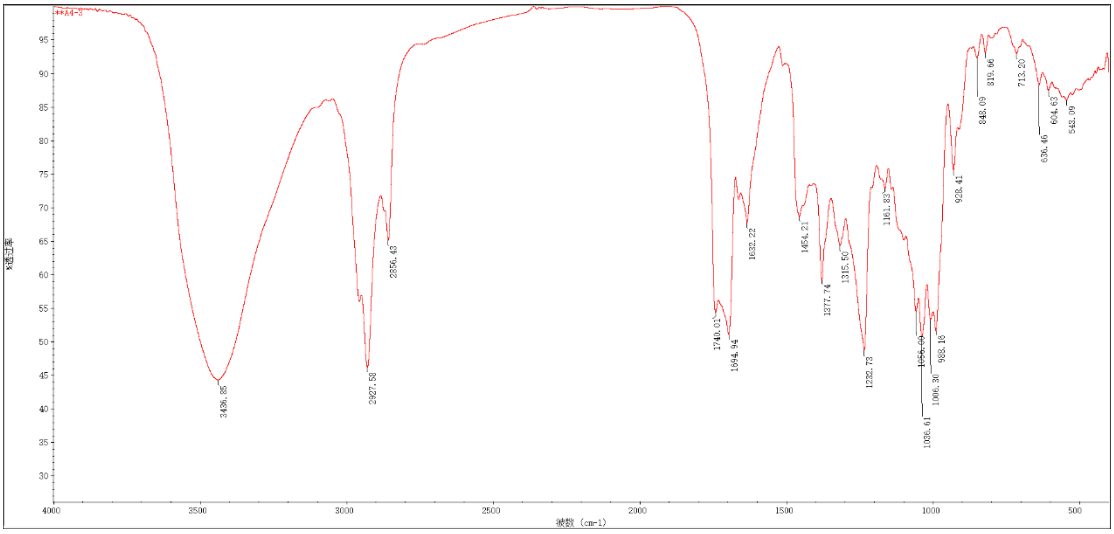

Figure S15. IR spectrum of **2**

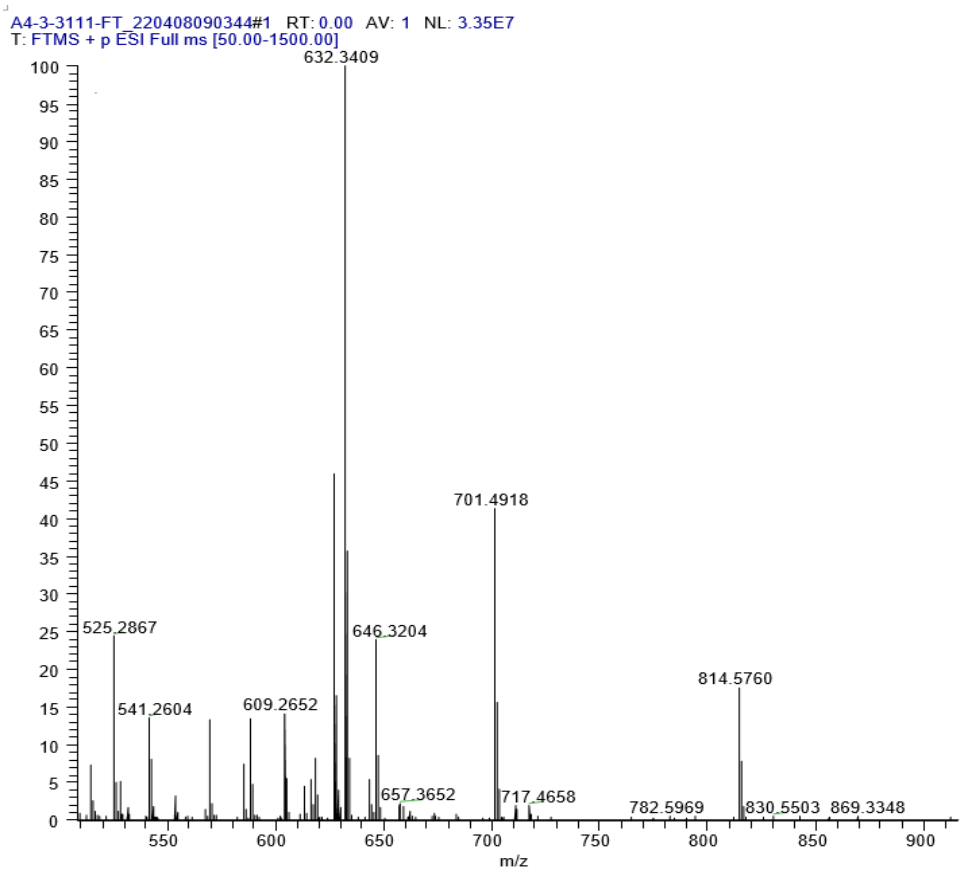

Figure S16. HRESIMS spectrum of **2**

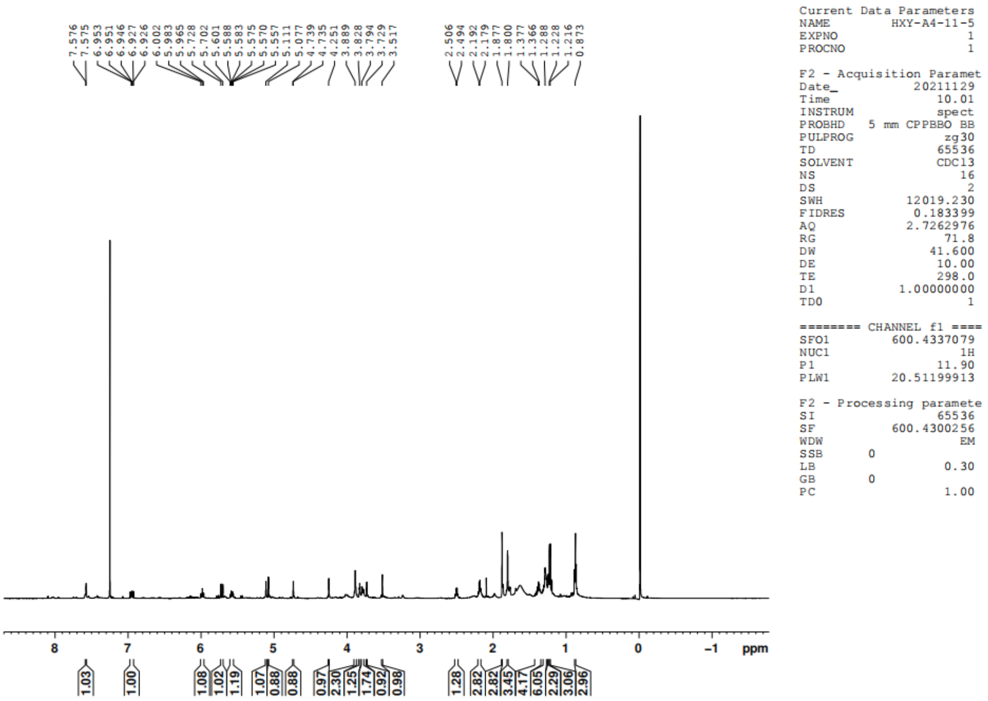

Figure S17. ^{1}H-NMR (600 MHz, $CDCl_3$) spectrum of **3**

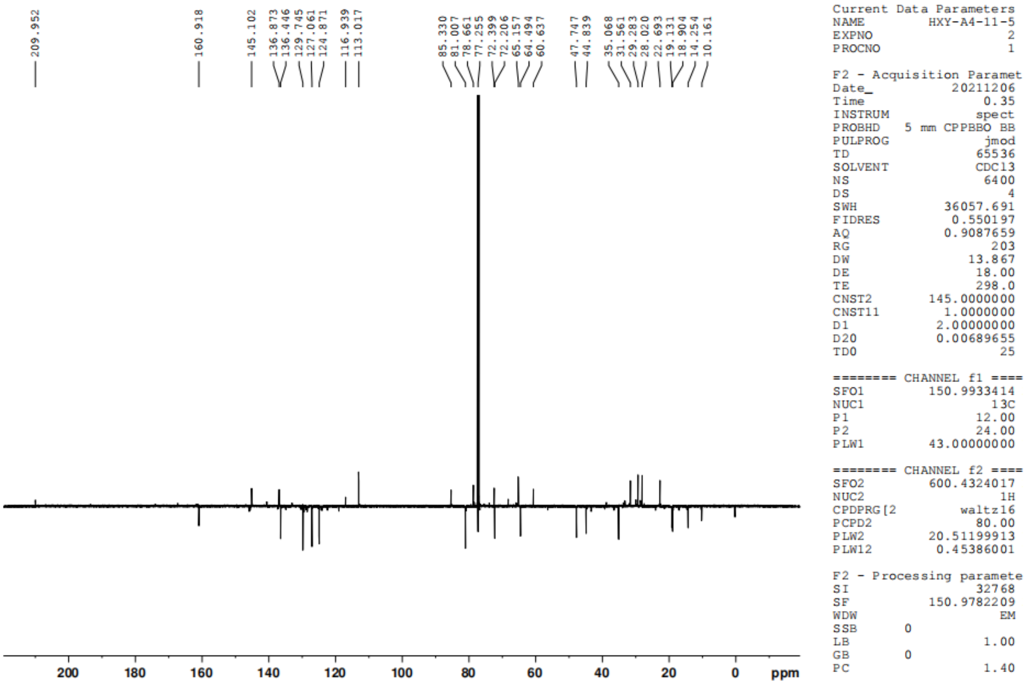

Figure S18. ^{13}C-APT (150 MHz, $CDCl_3$) spectrum of **3**

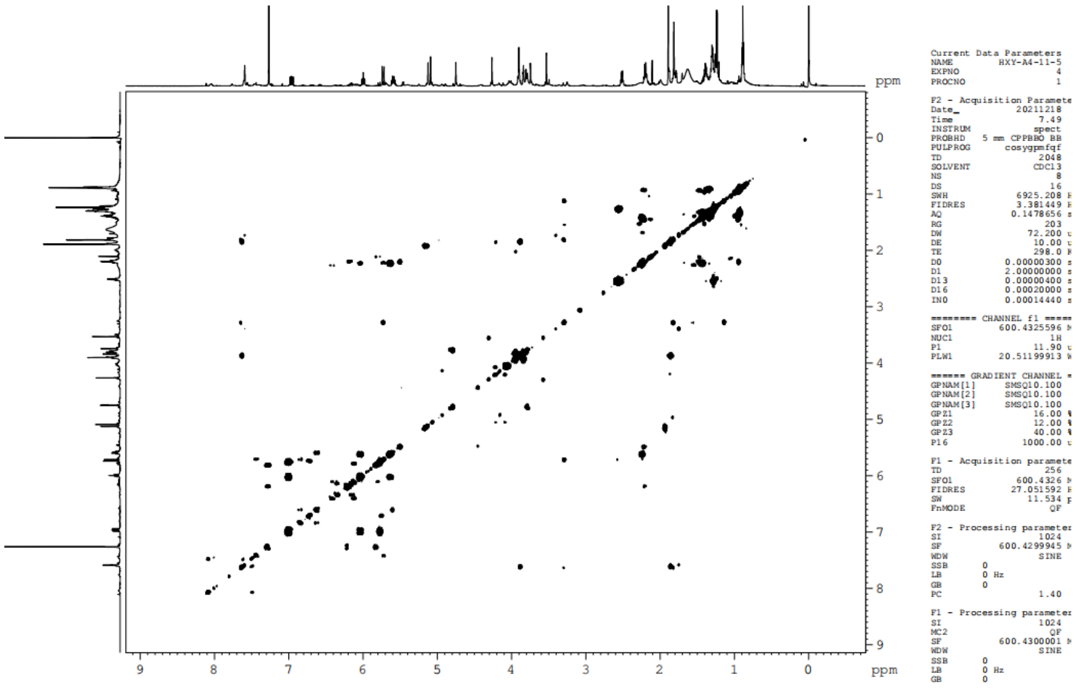

Figure S19. ^{1}H-^{1}H COSY ($CDCl_3$) spectrum of **3**

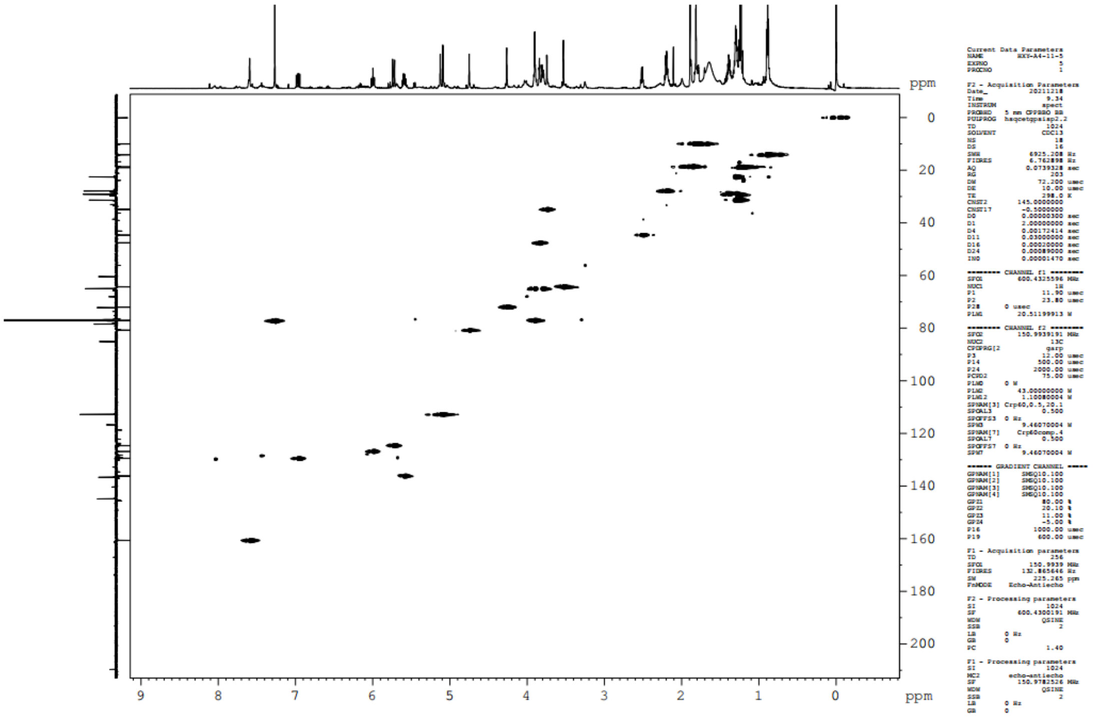

Figure S20. HSQC ($CDCl_3$) spectrum of **3**

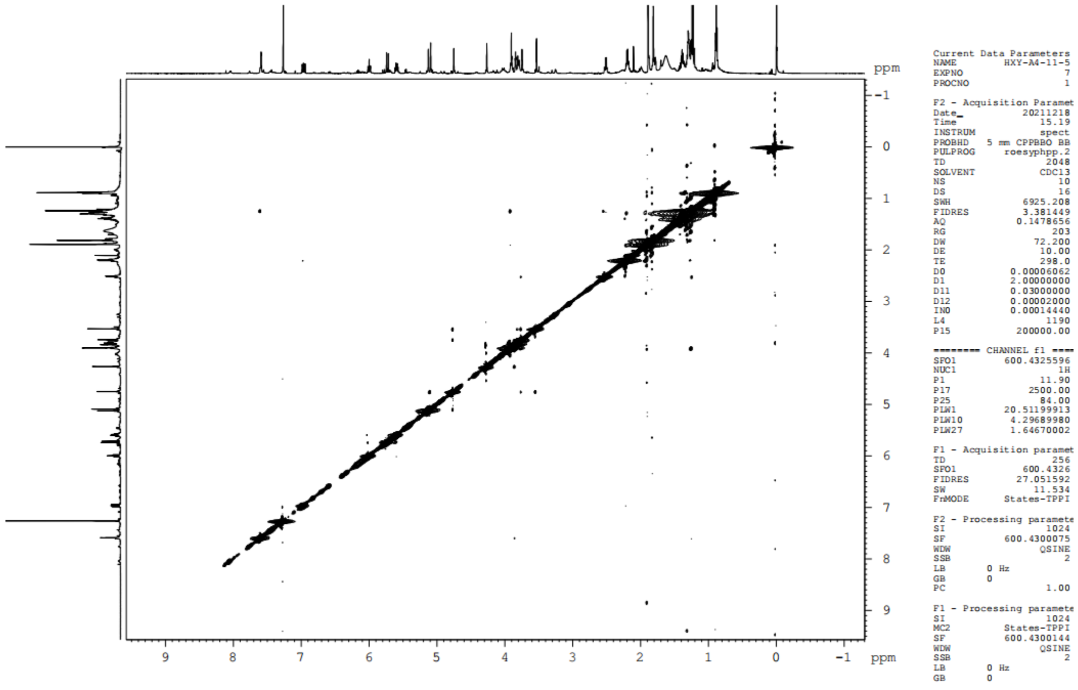

Figure S21. NOESY ($CDCl_3$) spectrum of **3**

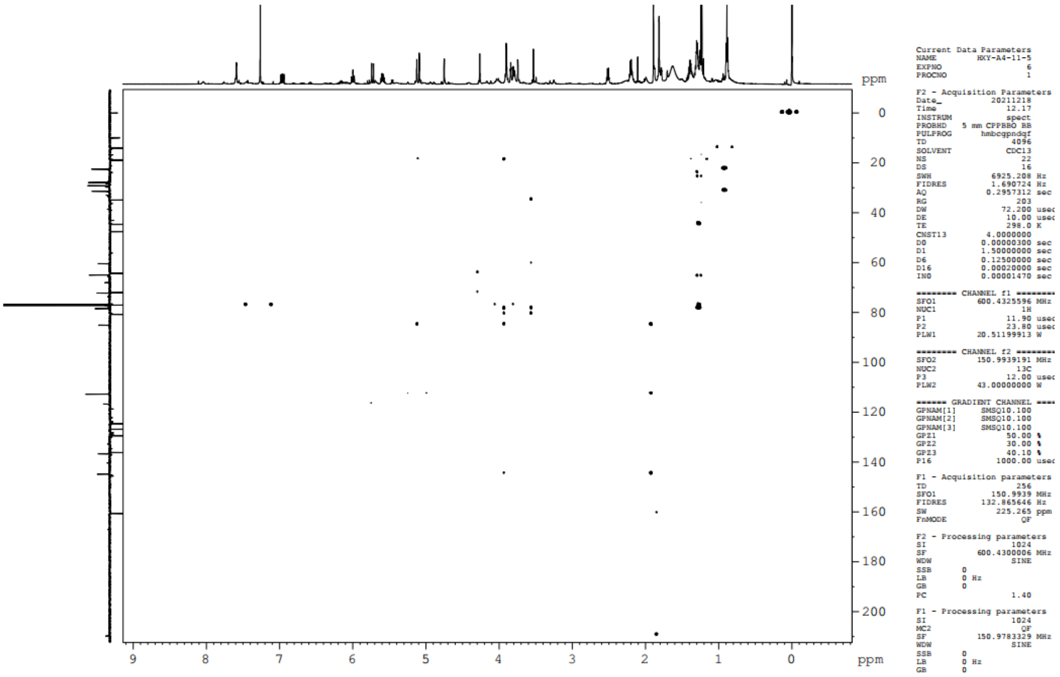

Figure S22. HMBC ($CDCl_3$) spectrum of **3**

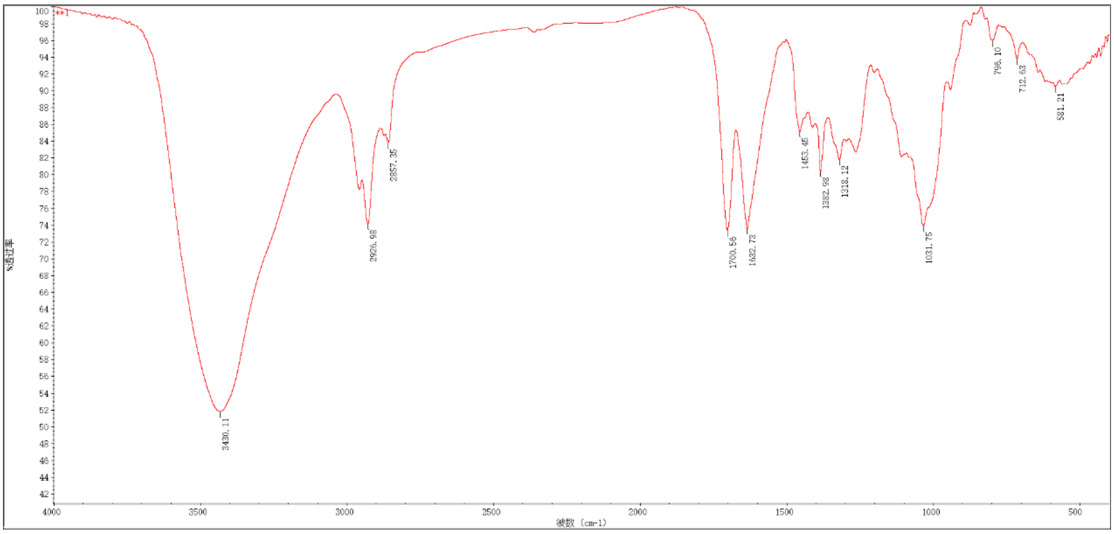

Figure S23. IR spectrum of **3**

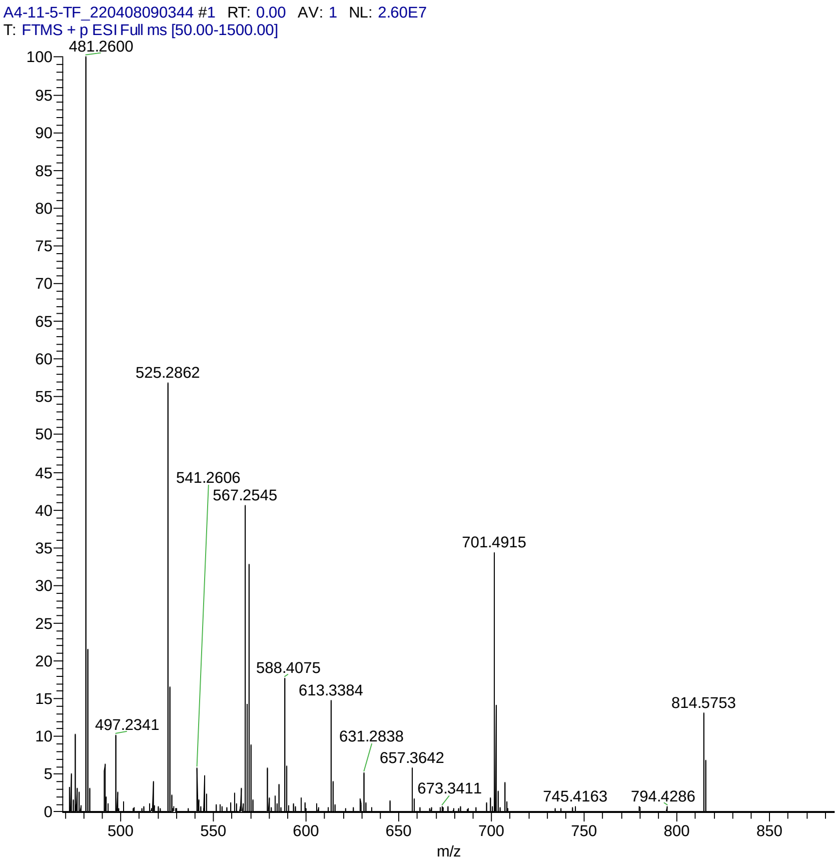

Figure S24. HRESIMS spectrum of **3**

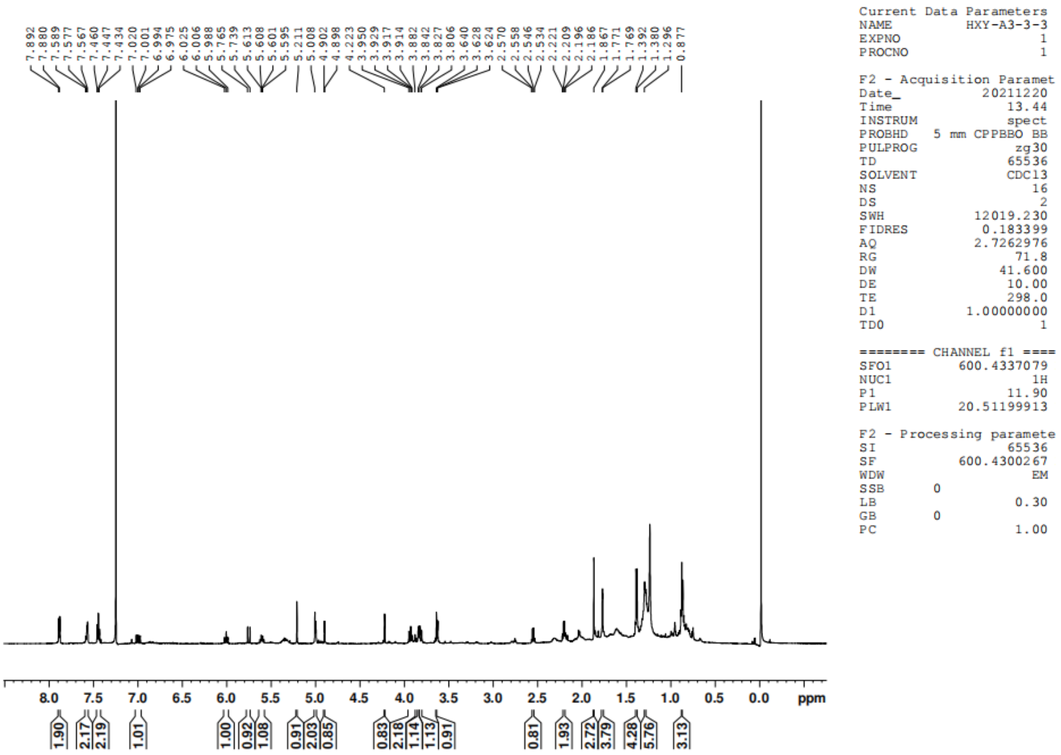

Figure S25. ^{1}H-NMR (600 MHz, $CDCl_3$) spectrum of **4**

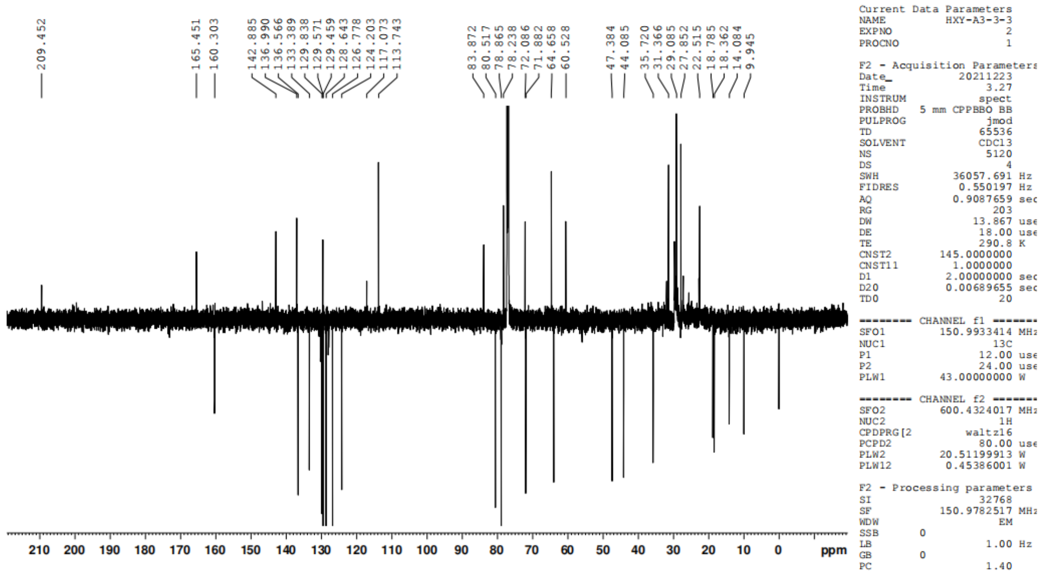

Figure S26. ^{13}C-APT (150 MHz, $CDCl_3$) spectrum of **4**

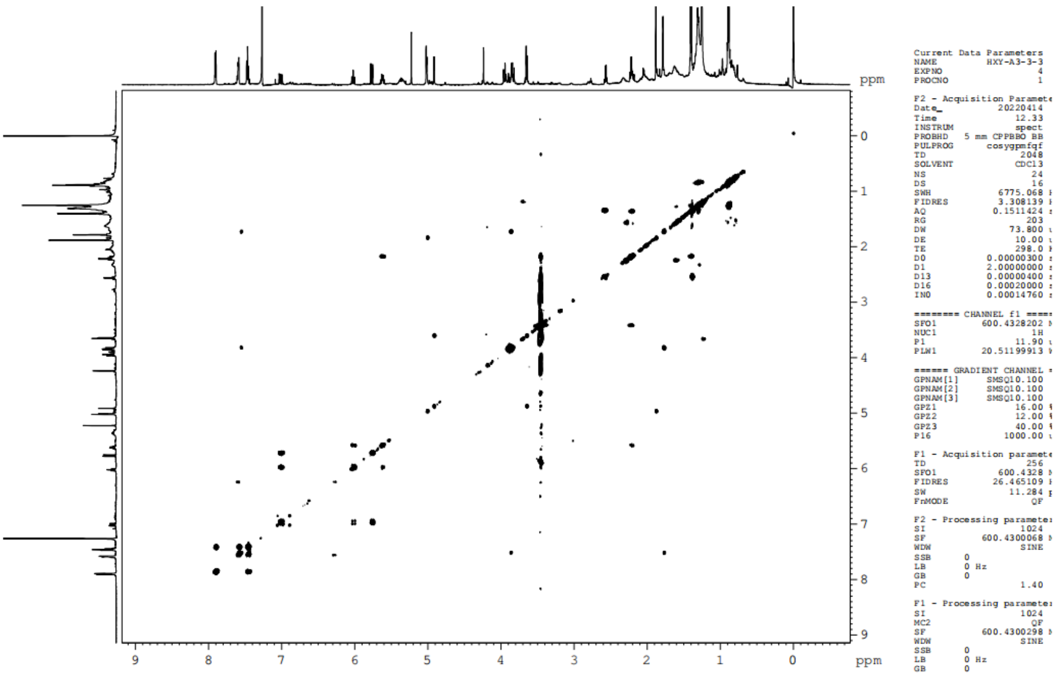

Figure S27. ^{1}H-^{1}H COSY ($CDCl_3$) spectrum of **4**

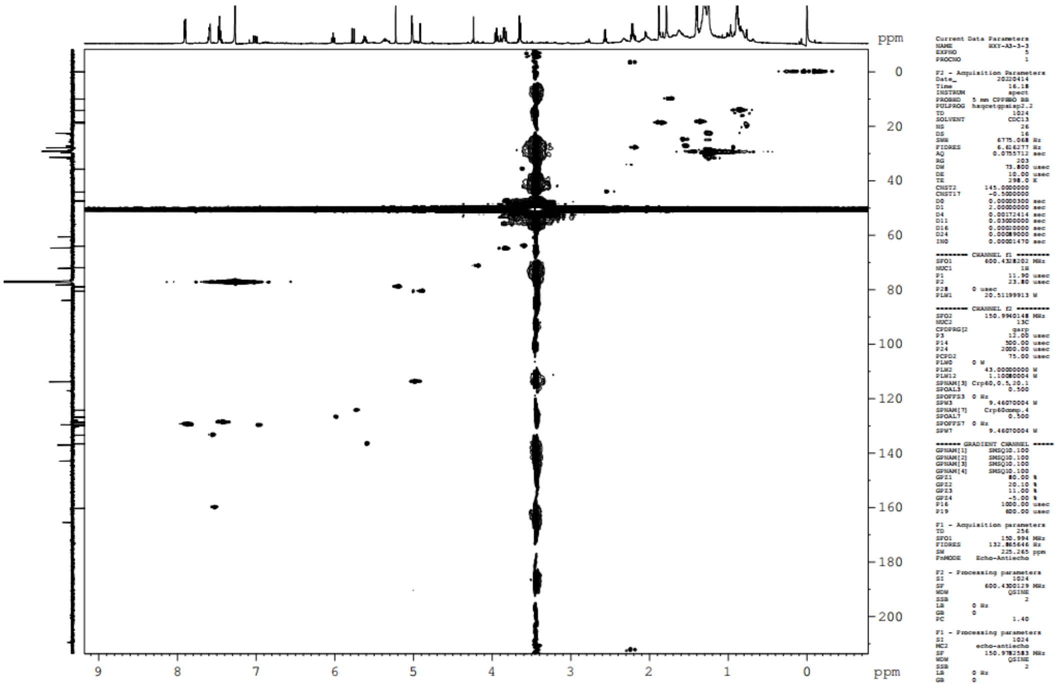

Figure S28. HSQC ($CDCl_3$) spectrum of **4**

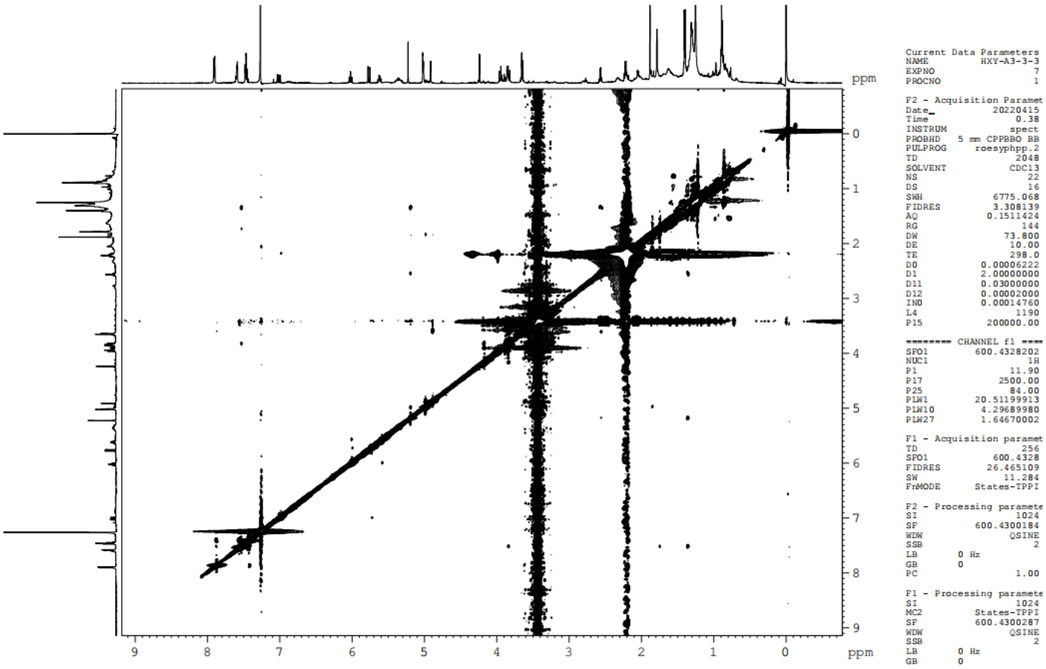

Figure S29. NOESY ($CDCl_3$) spectrum of **4**

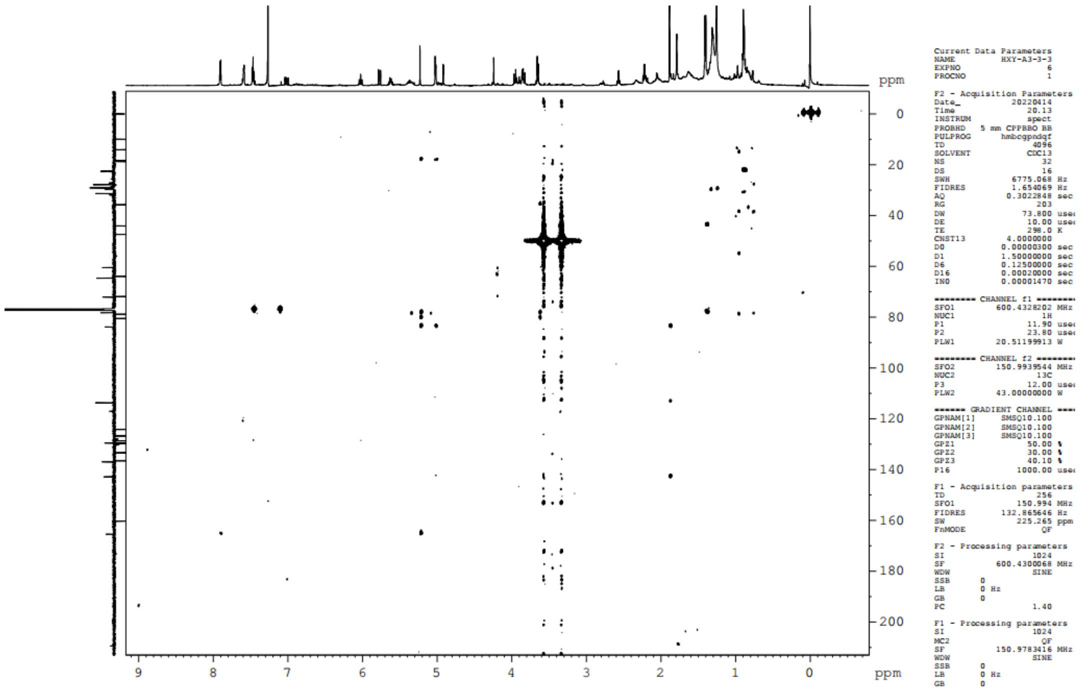

Figure S30. HMBC ($CDCl_3$) spectrum of **4**

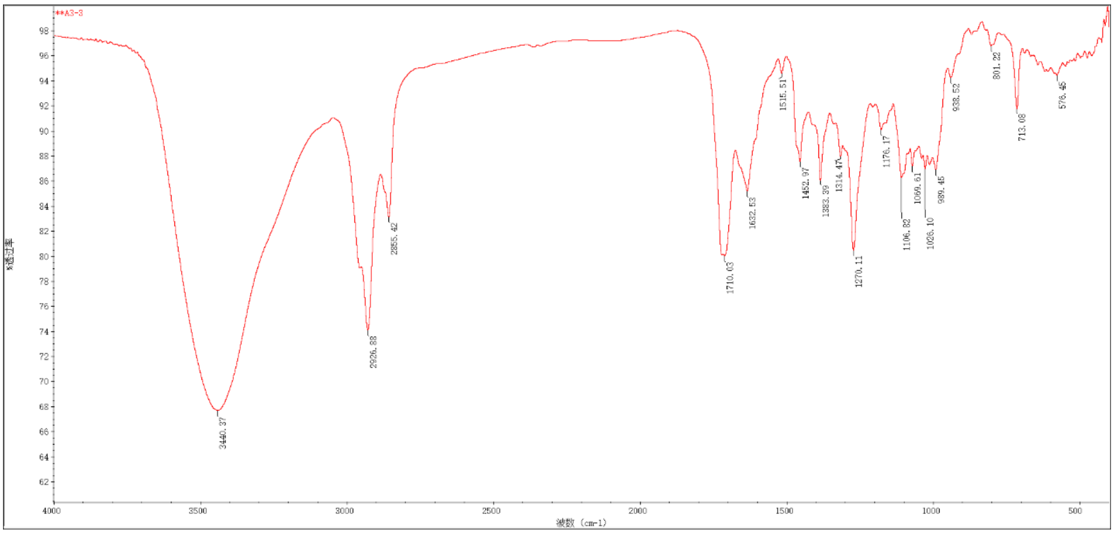

Figure S31. IR spectrum of **4**

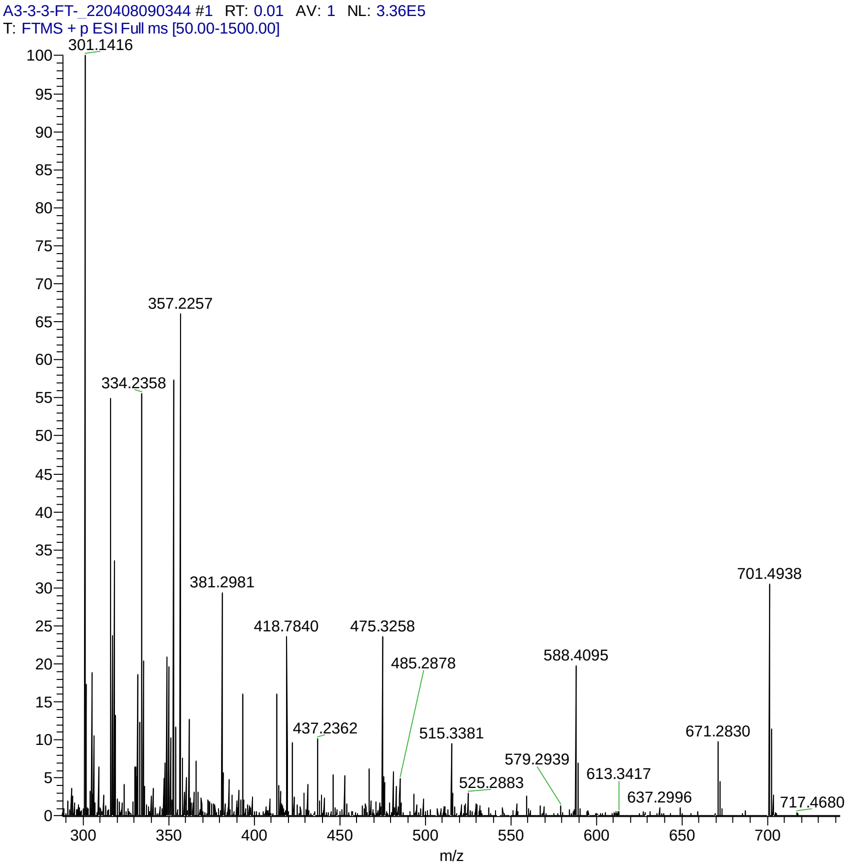

Figure S32. HRESIMS spectrum of **4**

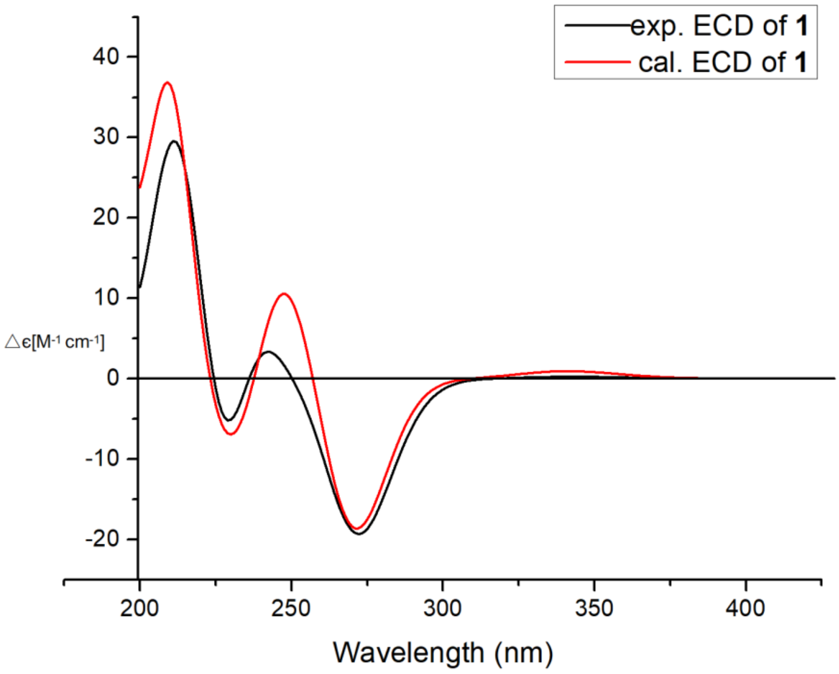

Figure S33. Experimental and calculated ECD spectra of **1**

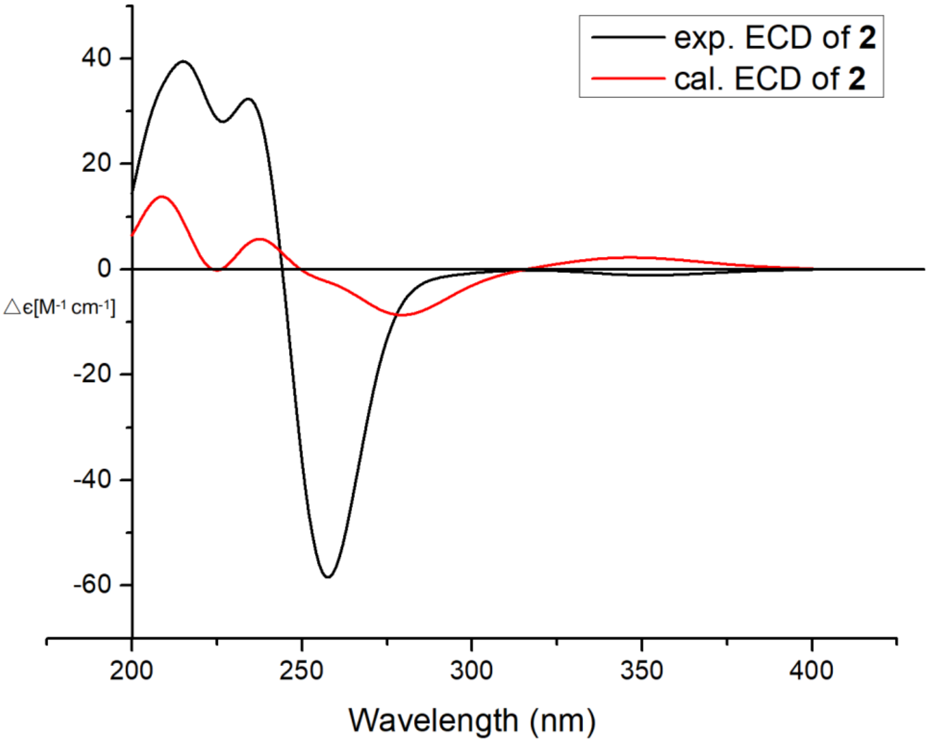

Figure S34. Experimental and calculated ECD spectra of **2**

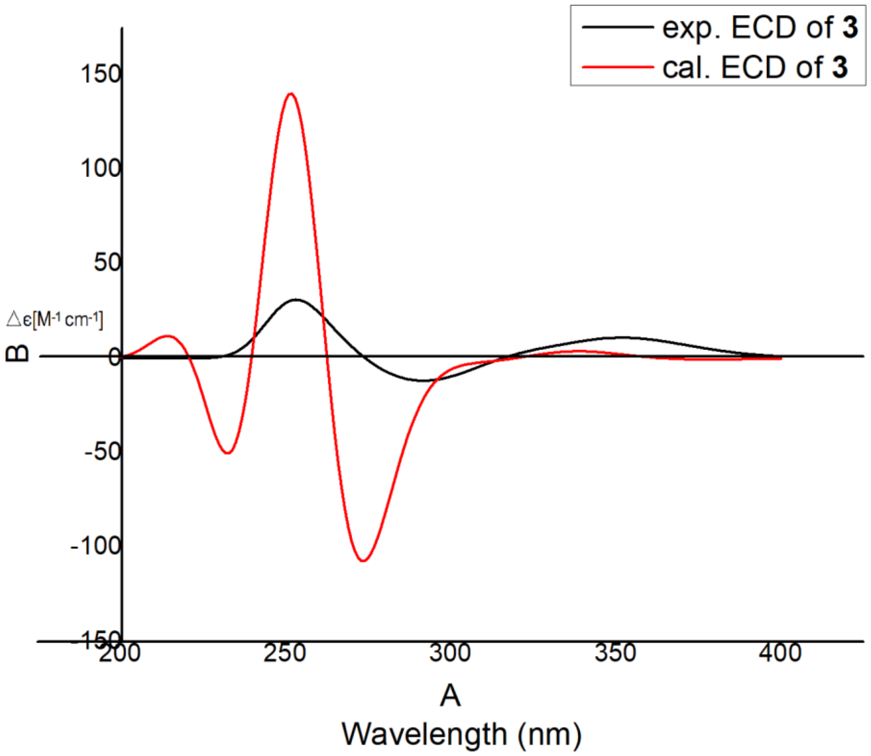

Figure S35. Experimental and calculated ECD spectra of **3**

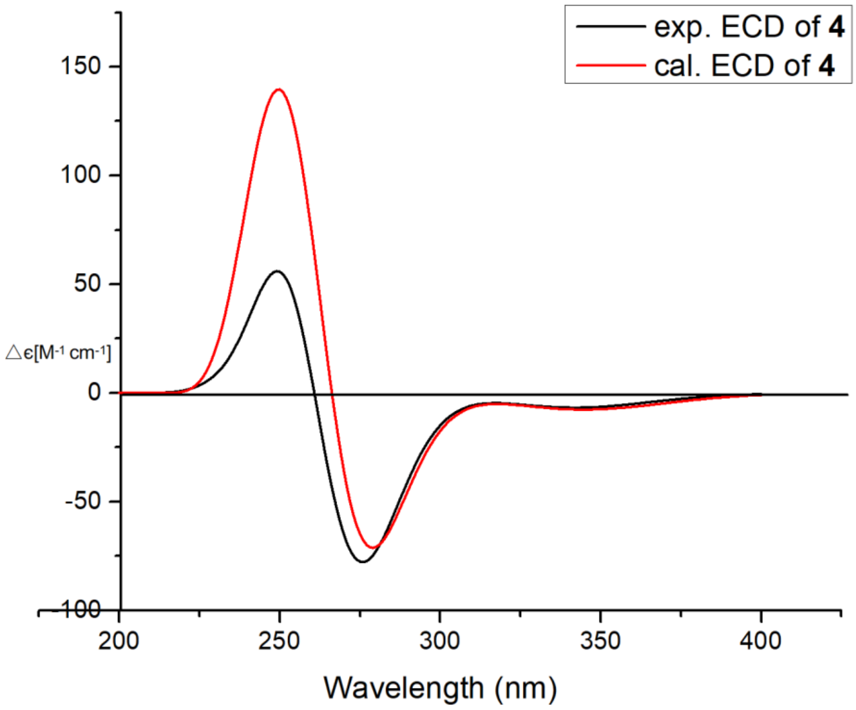

Figure S36. Experimental and calculated ECD spectra of **4**

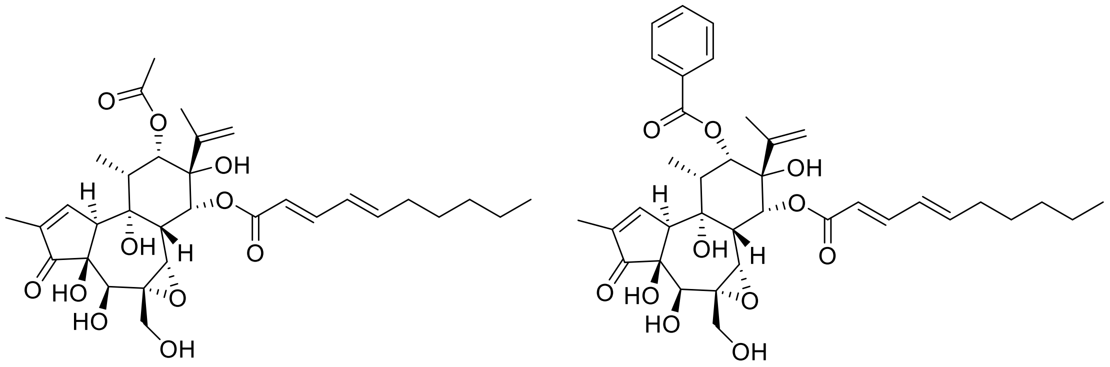

Figure S37. The structure of tianchaterpene A and tianchaterpene B.

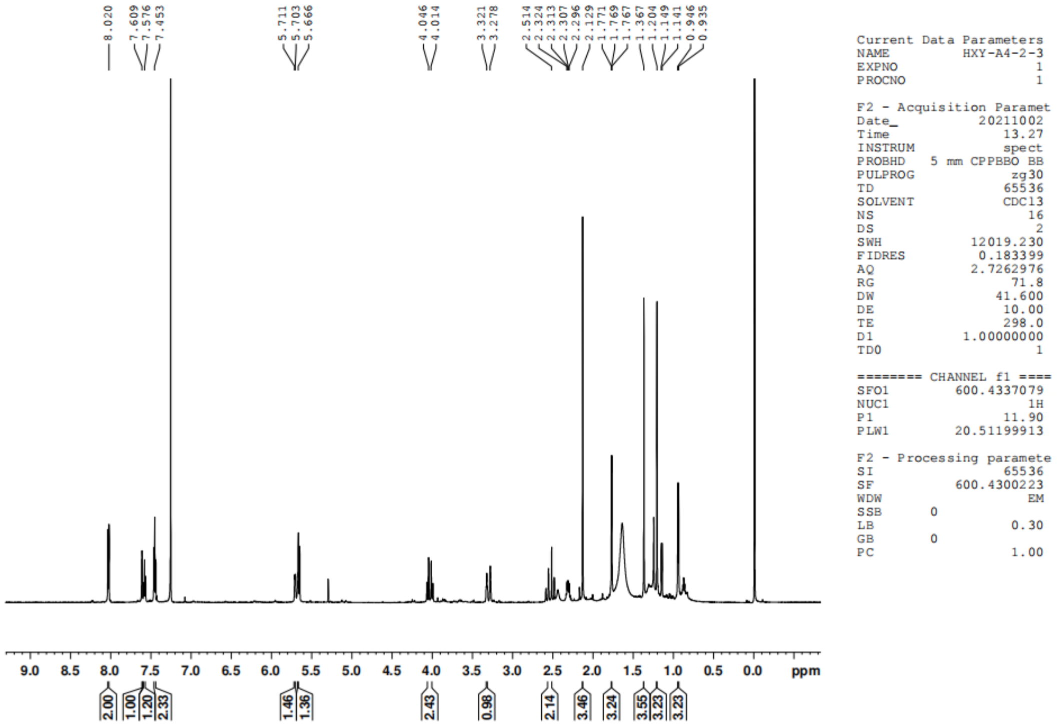

Figure S38. ^{1}H-NMR (600 MHz, $CDCl_3$) spectrum of **5**

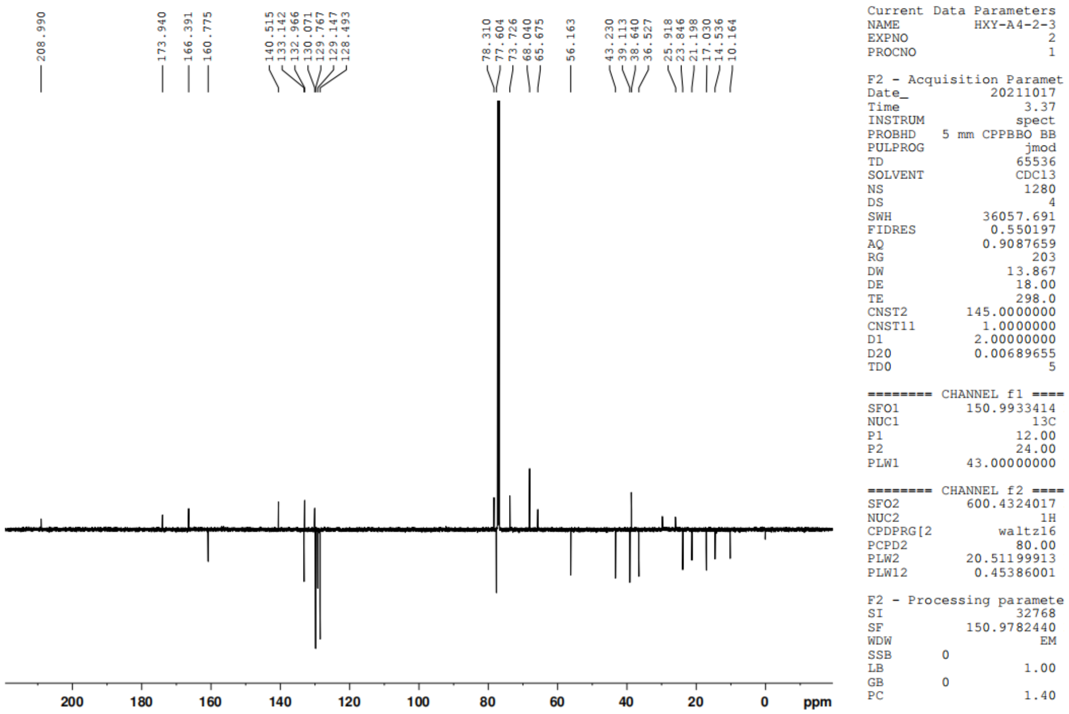

Figure S39. ^{13}C-APT (150 MHz, $CDCl_3$) spectrum of **5**

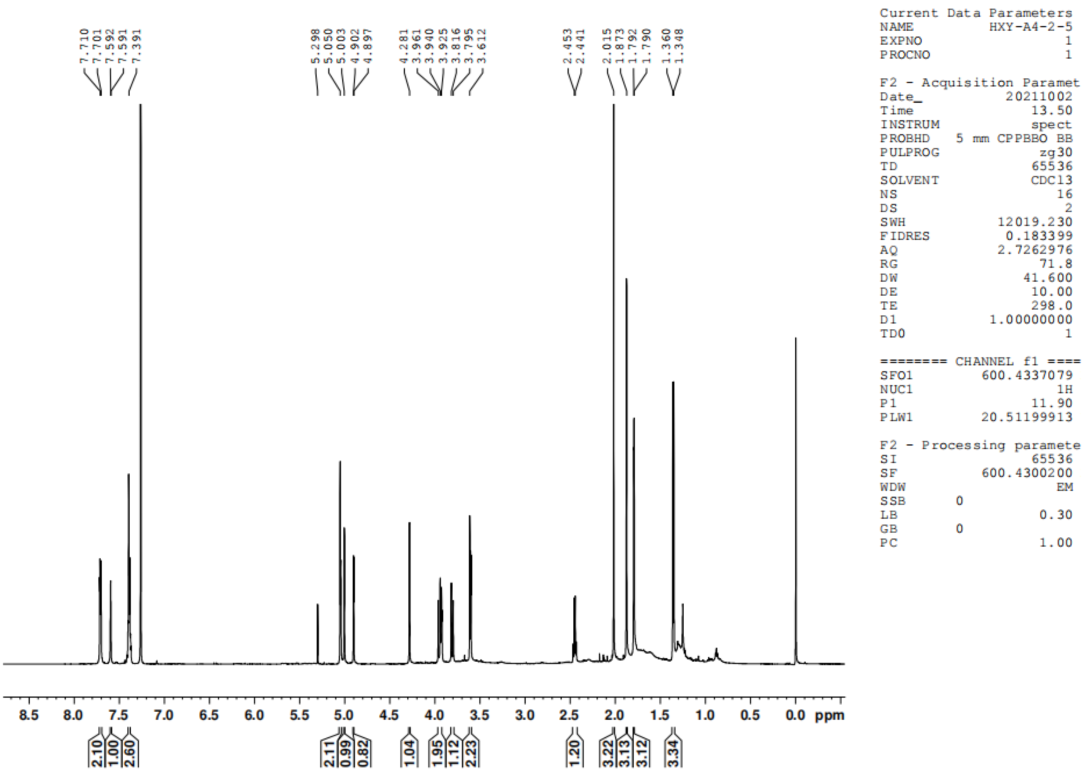

Figure S40. ^{1}H-NMR (600 MHz, $CDCl_3$) spectrum of **6**

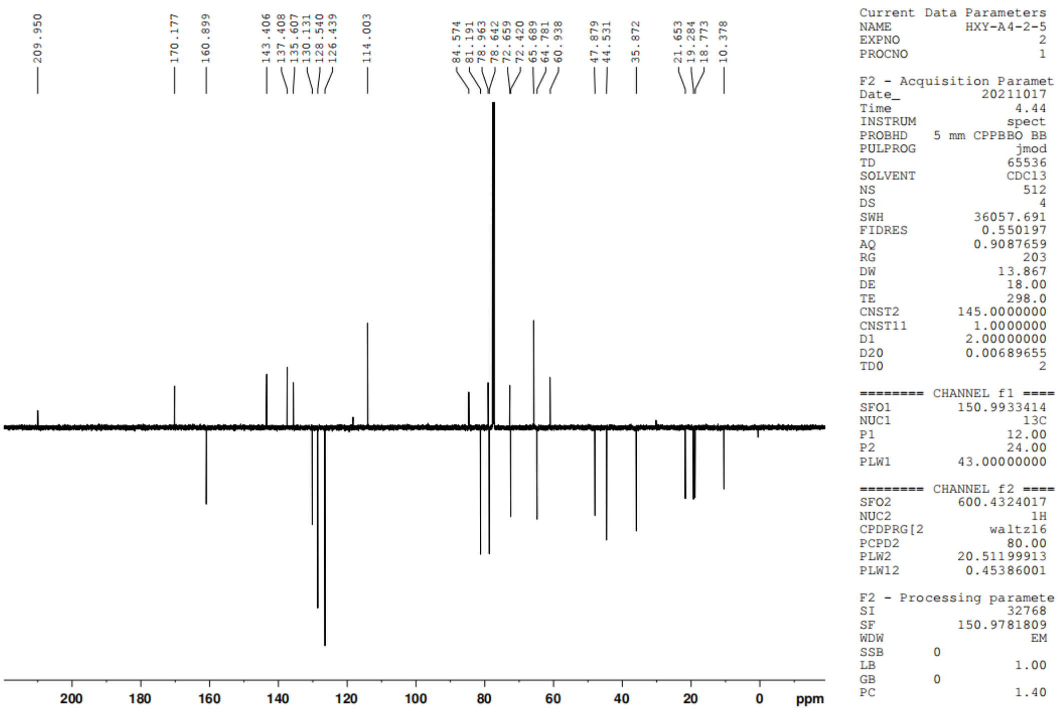

Figure S41. ^{13}C-APT (150 MHz, $CDCl_3$) spectrum of **6**

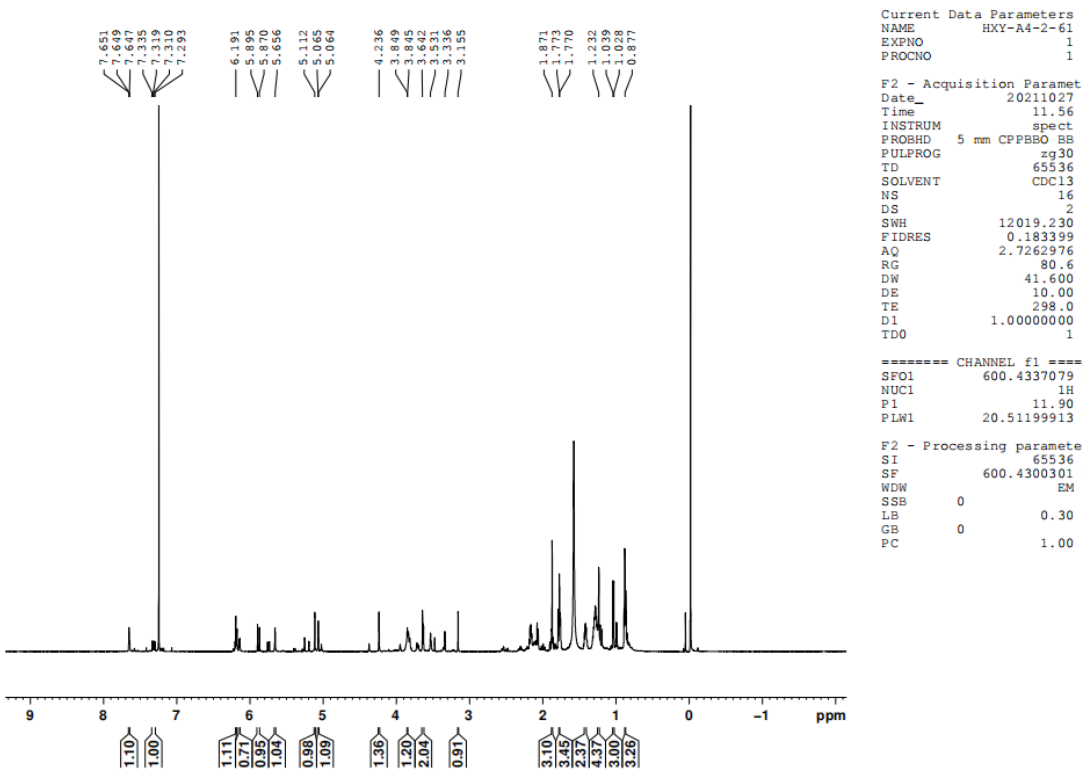

Figure S42. ^{1}H-NMR (600 MHz, $CDCl_3$) spectrum of **7**

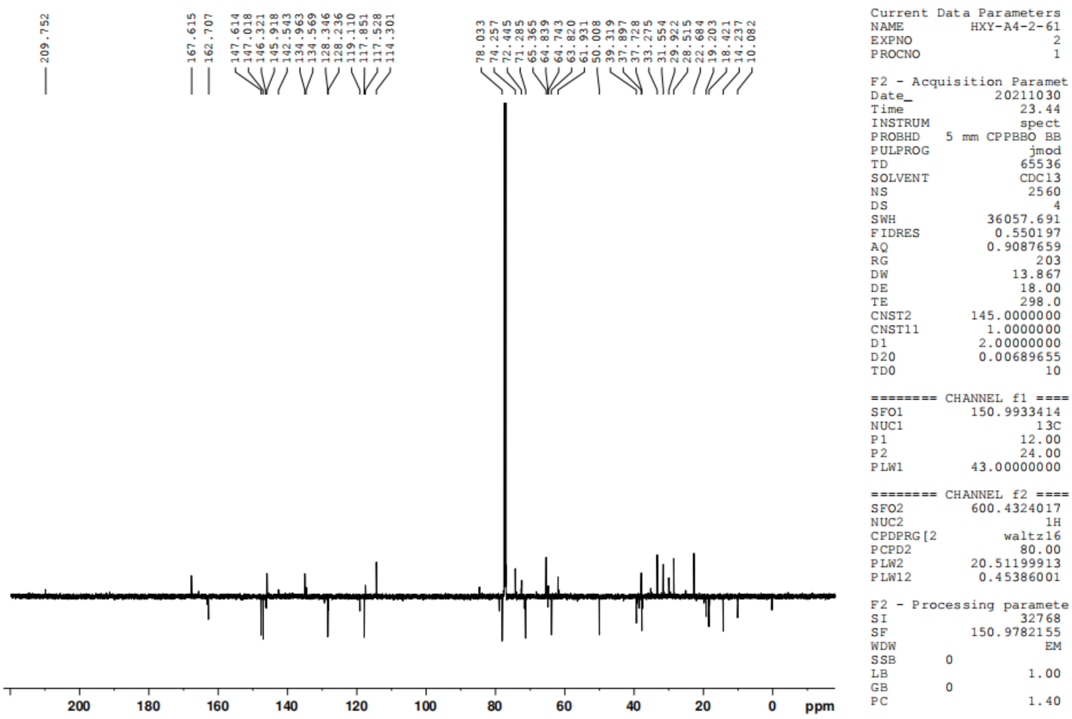

Figure S43. ^{13}C-APT (150 MHz, $CDCl_3$) spectrum of **7**

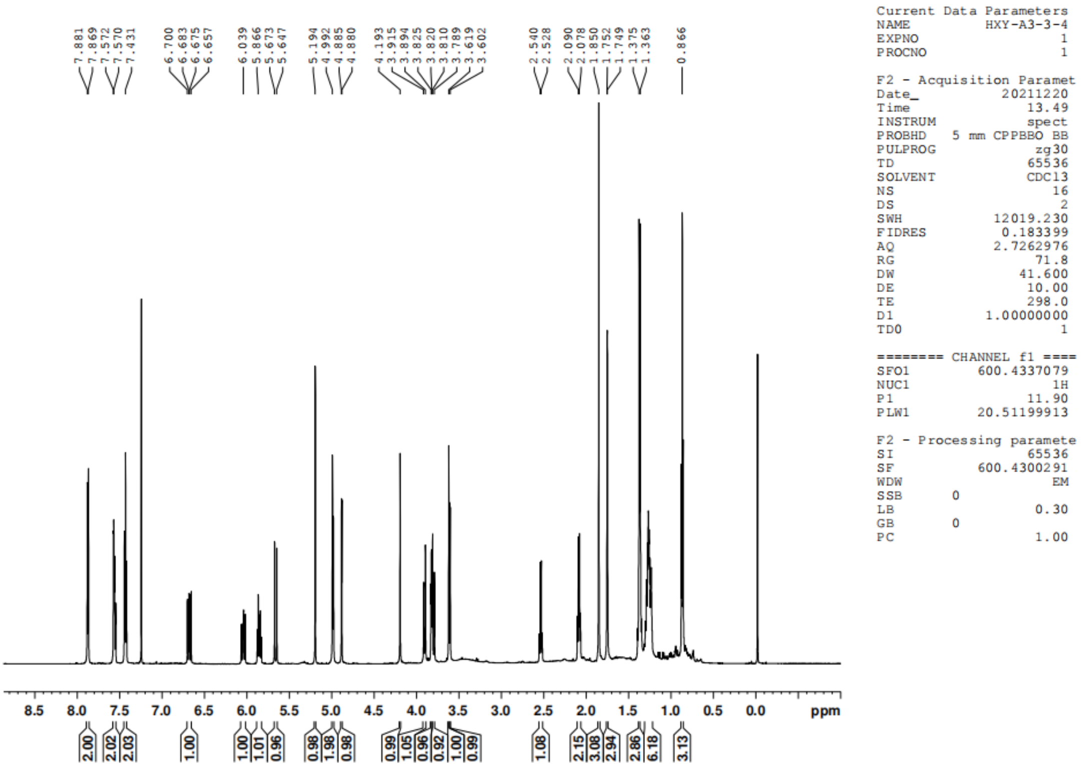

Figure S44. ^{1}H-NMR (600 MHz, $CDCl_3$) spectrum of **8**

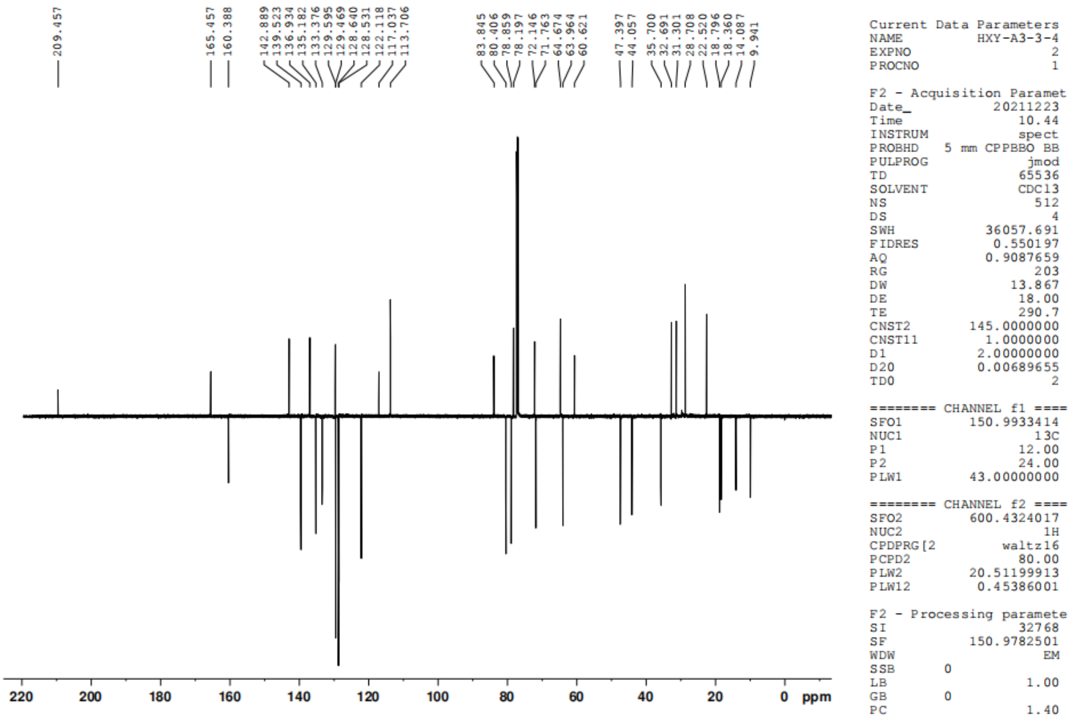

Figure S45. ^{13}C-APT (150 MHz, $CDCl_3$) spectrum of **8**

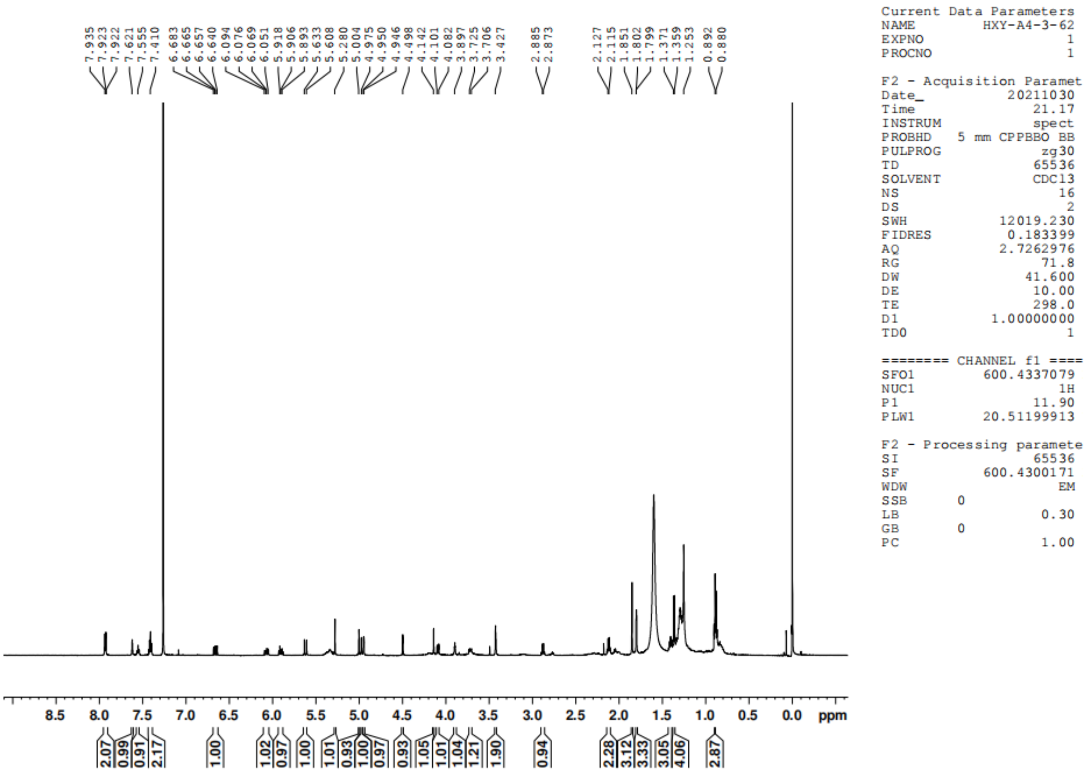

Figure S46. ^{1}H-NMR (600 MHz, $CDCl_3$) spectrum of **9**

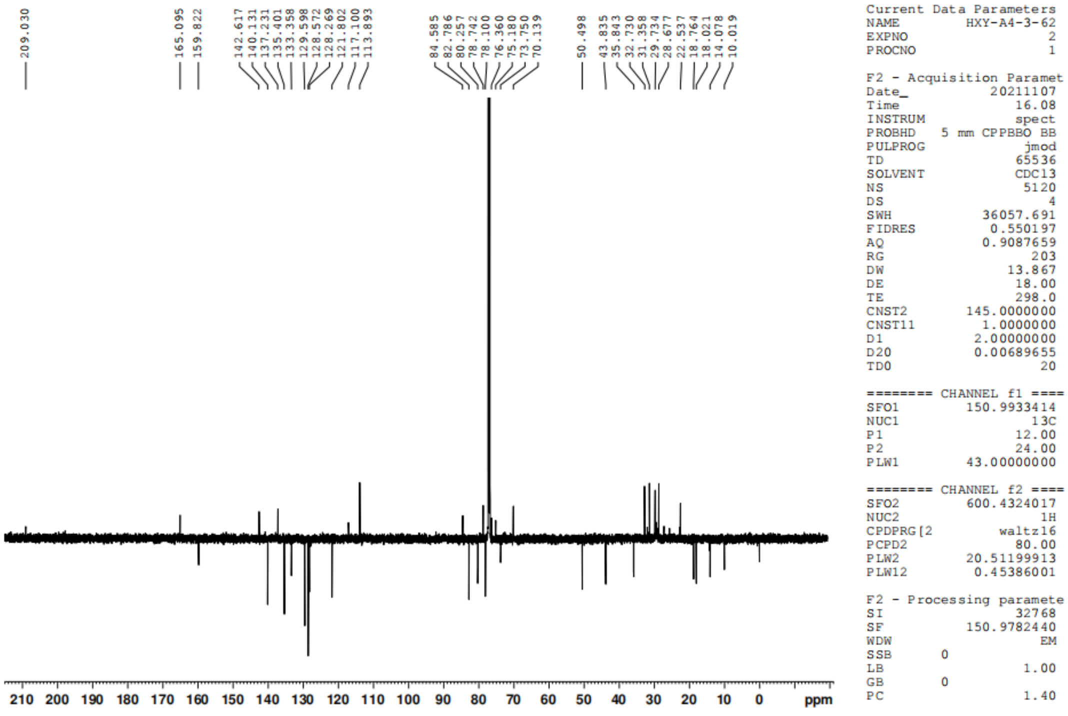

Figure S47. ^{13}C-APT (150 MHz, $CDCl_3$) spectrum of **9**

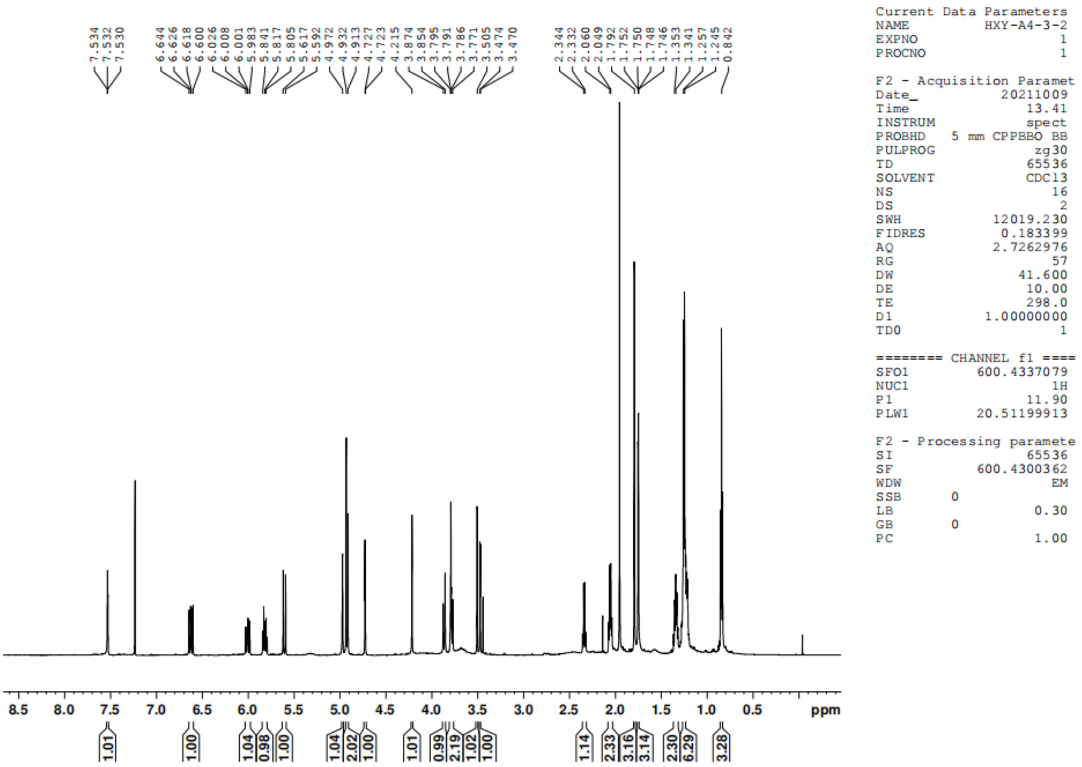

Figure S48. ^{1}H-NMR (600 MHz, $CDCl_3$) spectrum of **10**

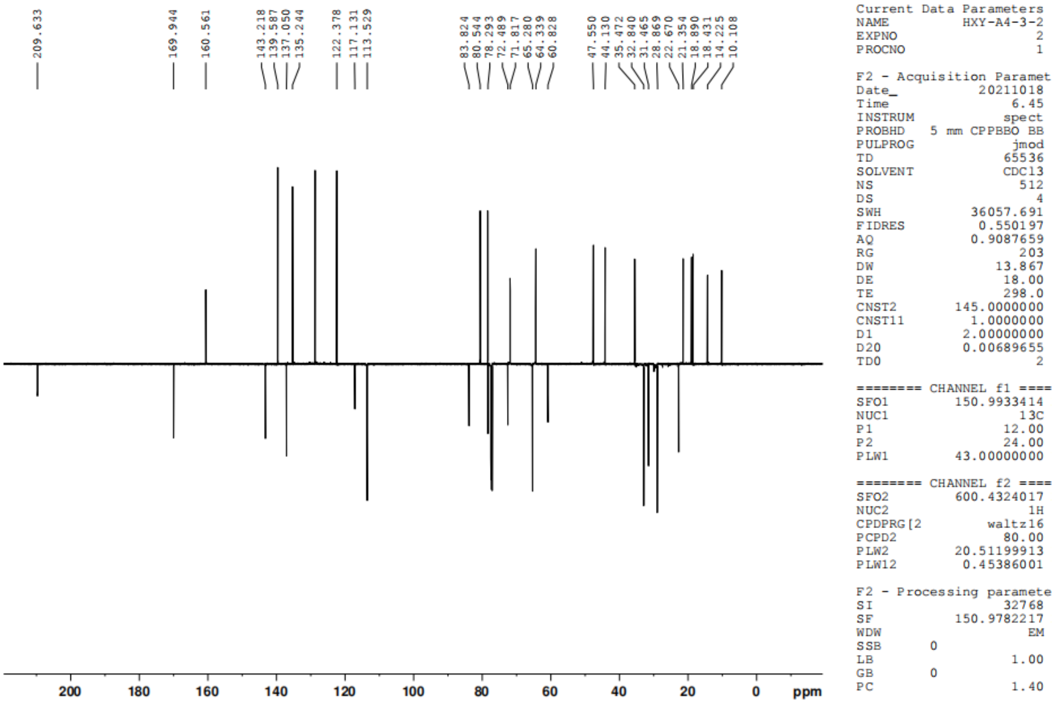

Figure S49. ^{13}C-APT (150 MHz, $CDCl_3$) spectrum of **10**

The figures of cytotoxicity data (against HGC-27) for the tested compounds **1-10**

Compound 1

Concentration(*μ*M)	$\bar{x}$ OD	%Viability ± STDEV ($\bar{v}$ ± s)
12.5	1.4065	14.4933 ±0.54
25	1.3885	15.5876 ±1.26
50	0.5385	67.2624 ±1.43
100	0.1285	92.1880 ±0.78
200	0.11	93.3127 ±0.89

Compound 2

Concentration(*μ*M)	$\bar{x}$ OD	$\bar{v}$ ± s
12.5	0.6200	51.1157 ±0.96
25	0.1630	87.1482 ±0.67
50	0.0860	93.2193 ±0.98
100	0.0835	93.4164 ±1.39
200	0.076	94.0077 ±2.27

Compound 3

Concentration(*μ*M)	$\bar{x}$ OD	$\bar{v}$ ± s
12.5	0.8865	30.1033 ±1.08
25	0.6695	47.2128 ±0.96
50	0.1155	90.8933 ±0.74
100	0.0875	93.1010 ±1.68
200	0.0730	94.2443 ±2.09

Compound 4

Concentration(*μ*M)	$\bar{x}$ OD	$\bar{v}$ ± s
12.5	0.6200	11.8505 ±1.31
25	0.1630	44.8474 ±0.92
50	0.0860	92.5491 ±1.65
100	0.0835	93.4164 ±1.67
200	0.0760	93.7712±2.08

Compound 5

Concentration(*μ*M)	$\bar{x}$ OD	$\bar{v}$ ± s
12.5	1.37	16.7123 ±2.15
25	1.3625	17.1682 ±1.31
50	0.315	80.8499 ±2.18
100	0.128	92.2184 ±1.09
200	0.1055	93.5862 ±0.77

Compound 6

Concentration(μM)	$\bar{x}$ OD	$\bar{v} \pm s$
12.5	1.271	22.7309 ±1.26
25	1.2805	22.1533 ±0.79
50	0.5155	68.6607 ±1.23
100	0.1235	92.4919 ±2.61
200	0.1065	93.5254 ±0.81

Compound 7

Concentration(μM)	$\bar{x}$ OD	$\bar{v} \pm s$
12.5	1.416	13.9157 ±0.29
25	1.1945	27.3816 ±2.01
50	0.5059	69.0255 ±0.94
100	0.12	92.7047 ±1.09
200	0.128	92.2184 ±0.94

Compound 8

Concentration(μM)	$\bar{x}$ OD	$\bar{v} \pm s$
12.5	0.9635	41.4250 ±0.92
25	0.4675	71.5788 ±1.07
50	0.112	93.1911 ±1.37
100	0.0925	94.3766±2.38
200	0.0895	94.5589 ±1.63

Compound 9

Concentration(μM)	$\bar{x}$ OD	$\bar{v} \pm s$
12.5	1.063	35.3760 ±0.68
25	0.654	60.2407 ±2.39
50	0.123	92.5223±2.11
100	0.094	94.2854 ±1.97
200	0.087	94.7109 ±1.64

Compound 10

Concentration(μM)	$\bar{x}$ OD	$\bar{v} \pm s$
12.5	0.962	41.5162 ±0.91
25	0.098	94.0422 ±1.25
50	0.0835	94.9237 ±1.09
100	0.077	95.3189 ±0.98
200	0.0785	95.2277 ±1.67
